# Impact of peripheral circadian misalignment and alcohol on the resiliency of intestinal barrier and microbiota

**DOI:** 10.1080/19490976.2025.2509281

**Published:** 2025-06-04

**Authors:** Laura Tran, Maliha Shaikh, Phillip A. Engen, Ankur Naqib, Dulce M. Frausto, Vivian Ramirez, Malia Gasteier, Zlata Bogin, Kristi Lawrence, Lijuan Zhang, Shiwen Song, Stefan J. Green, Faraz Bishehsari, Christopher B. Forsyth, Ali Keshavarzian, Garth R. Swanson

**Affiliations:** a Rush Center for Integrated Microbiome and Chronobiology Research, Rush University Medical Center, Chicago, IL, USA; b Genomics and Microbiome Core Facility, Rush University Medical Center, Chicago, IL, USA; c Department of Pathology, GoPath Global Pathology Service, Buffalo Grove, IL, USA; d Department of Internal Medicine, Rush University Medical Center, Chicago, IL, USA; e Gastroenterology Research Center, Division of Gastroenterology, Hepatology & Nutrition, Department of Internal Medicine, University of Texas, Houston, TX, USA; f MD Anderson Cancer Center-UT Health Houston Graduate School of Biomedical Sciences, Texas Medical Center, Houston, TX, USA; g Departments of Physiology, Rush University Medical Center, Chicago, IL, USA; h Department of Anatomy and Cell Biology, Rush University Medical Center, Chicago, IL, USA; i Department of Medicine, Medical University of South Carolina, Charleston, SC, USA

**Keywords:** Circadian disruption, intestinal permeability, microbiota, colonic organoid

## Abstract

Circadian organization is involved in many gastrointestinal tract (GIT) functions such as the maintenance of intestinal barrier integrity. There is compelling evidence that perturbation of the circadian clock decreases intestinal epithelial cells’ resiliency to alcohol-induced injury. One of the most common causes of circadian misalignment is wrong-time eating (largest meal at dinner) in modern societies. Yet, few studies have examined the importance of peripheral circadian rhythms of the GIT to alcohol consumption. Eating patterns during physiologic rest time, defined as wrong-time eating (WTE), misalign the peripheral circadian clock of the GIT and the body’s central clock. This study aims to fill this knowledge gap by testing the hypothesis that: (1) WTE worsens alcohol-induced disruption of intestinal barrier integrity and (2) decreased intestinal barrier resiliency to alcohol effects by WTE-disrupted circadian is, at least in part, due to microbiota dysbiosis. Alcohol (20% v/v) and a restricted timed-food paradigm were administered to PERIOD2 luciferase (PER2:LUC) reporter BL/6 mice for 10 weeks. Intestinal barrier integrity, intestinal (stool) microbiota, and microbial metabolites (cecal-derived) were examined. Peripheral circadian misalignment exacerbated alcohol-induced disruption of intestinal barrier integrity (tight junctional proteins) leading to increased intestinal permeability (*p* < 0.05). In addition, alcohol consumption changed the intestinal microbiota community, decreasing beneficial short-chain fatty acid-producing taxa. Further, we recapitulated the in vivo phenotype in a colonic organoid model and demonstrated that microbial metabolites from circadian-disrupted, alcohol-fed mice mediate decreased resiliency of intestinal epithelial barrier function. Peripheral circadian misalignment through food timing decreases the resiliency of the intestinal barrier to alcohol-induced injury and this effect is mediated through dysbiotic microbiota metabolites.

## Introduction

Circadian rhythms are endogenous 24-h oscillations controlled by biological clocks that are involved in functions such as the sleep-wake cycle and multiple physiologic activities.^1^ While the central circadian clock is entrained by light, the peripheral circadian clock has autonomous circadian control and is entrained by other specific factors.^2^ In the gastrointestinal tract (GIT), the main entrainer, or zeitgeber, is the time of food consumption.^3^ Therefore, while abnormal exposure to light as in shift work disrupts central circadian rhythms, altered eating patterns during physiologic rest time, defined as wrong-time eating (WTE), disrupts peripheral circadian rhythms and misaligns the peripheral circadian clock of the GIT and the body’s central clock.

Modern lifestyle has led to an increased prevalence of a 24-h society where several aspects of the modern “Western” societies commonly disrupt circadian rhythms. This includes shift work, increased nighttime light exposure (e.g., use of light emitting devices while in bed, bright street lights), social jet lag (different sleep patterns on work days, and free days), frequent long-haul travels over two time zones, irregular eating patterns (e.g., eating at different times each day, eating late at night, eating large meals close to biological rest time), and high-fat diet composition.^4–11^ While all these lifestyle features contribute to circadian disruption, shift work has the most impactful and dramatic effect on the central circadian clock.^12^ However, since irregular eating patterns are extremely common then it is highly plausible that “wrong-time” eating could be even more impactful than shift work.

The GI tract is the largest interface between the host and environment and is one of the primary gatekeepers to protect the host from the deleterious effects of environmental factors and maintain a healthy state; conversely, if the “gatekeeping” function of the GI tract is compromised, it could be the site for injurious environmental factors to trigger and/or worsen disease states. The intestinal epithelial barrier is one of the critical elements of this gatekeeping function of the GI tract.^13^ A network of cell-cell junctions including tight junction proteins, adherens junctions, and desmosomes maintains this barrier. Disruption of this barrier can result in increased permeability and “leakiness” of the intestinal barrier which allows luminal toxins, bacteria, and their metabolites such as endotoxins (i.e., lipopolysaccharide [LPS]) to leave the gut lumen and enter the bloodstream to promote inflammation-mediated diseases and mediate organ damage.^14^

One environmental factor that can disrupt intestinal barrier integrity and promote endotoxemia is unhealthy alcohol consumption.^15^ Unhealthy alcohol consumption has been associated with a wide range of harmful health effects. Indeed, alcohol use disorder is associated with chronic liver disease, pancreatitis, muscle/bone disorders, and brain disorders with cognitive decline.^16,17^ Inflammation is the common underlying mechanism of alcohol-induced organ damage. We and others have shown that the primary source of this inflammatory state in the gut is alcohol-induced disruption of intestinal barrier integrity.^18^ Intriguingly, not all patients with alcohol use disorder develop organ damage suggesting unhealthy alcohol consumption is required but not sufficient to cause organ damage and additional factor(s) is required for unhealthy alcohol consumption to cause severe enough disruption of the intestinal barrier to initiate and sustained enough inflammatory state to cause organ damage.^19^ One such environmental factor is disrupted circadian homeostasis because we and others have shown that disrupted circadian can cause microbiota dysbiosis and intestinal leak.^20,21^

Studies evaluating the potential impact of disrupted circadian on the GI tract and intestinal barrier have primarily focused on central circadian disruption. In rodents, environmental central circadian disruption models (e.g., chronic phase shifts of the light/dark cycle).^21^ Similarly, genetic disruption through clock gene mutations (e.g., *Clock*^Δ19/Δ19^) in mice has worsened intestinal permeability that exacerbates alcohol-induced gut leakiness as well as dysbiosis (resulting in lower microbial diversity) in the presence of a stressor such as alcohol.^22,23^

In addition, knock-out models of the core clock machinery with Per2, Reverb-a, and Bmal1 have all shown the circadian clock mediates colonic inflammation, dysbiosis, and decreased production of bacterial-derived metabolites like short-chain fatty acids.^24–26^ In humans, alcohol consumption induces a systemic pro-inflammatory response and harms gut barrier integrity in night workers with circadian misalignment as well as causes dysbiosis of the gut microbiota.^12,27^

Yet, disruption of the intestinal circadian clock might be more relevant in the regulation of intestinal barrier integrity. However, there are very limited studies to determine whether disruption of intestinal circadian homeostasis by wrong-time eating can negatively impact intestinal barrier function and whether it can worsen alcohol-induced gut leak and endotoxemia. Our study aimed to fill this knowledge gap by utilizing both *in vivo* animal models and *ex vivo* organoid models.

## Results

Previously, we have shown that central circadian disruption through chronic LD shifting can increase the variability of the circadian period and phase in colonic tissue and organoids before altering food timing to impact peripheral colonic circadian rhythms.^21^ Food timing may elicit peripheral circadian phase-shifting effects and act as an environmental entrainer.^28^

In this study, to establish a phenotype of peripheral circadian disruption, we sought to administer the PER2:LUC mice under a food restriction paradigm defined with either day- (rest-phase= “wrong-time”, 7 AM) or nighttime (active-phase= “right-time”, 7 PM) access to food for 12 h for 5 consecutive weekdays followed by a 2-day *ad-libitum* access to food to mimic human conditions on the weekend. This food timing paradigm represents abnormal eating patterns that have been seen to shift the peripheral clocks in organs such as the liver and colon.^29^ In addition, the mice were maintained in a 12:12 cycle that followed the standard 12 h light (during the active phase, 7PM) and 12 h dark (during the rest phase). Together, these components formed the basis of our right-time, wrong-time experimental paradigm.

### Impact of wrong-time eating (WTE) and alcohol on peripheral (colon) circadian rhythm

**Figure 1.**
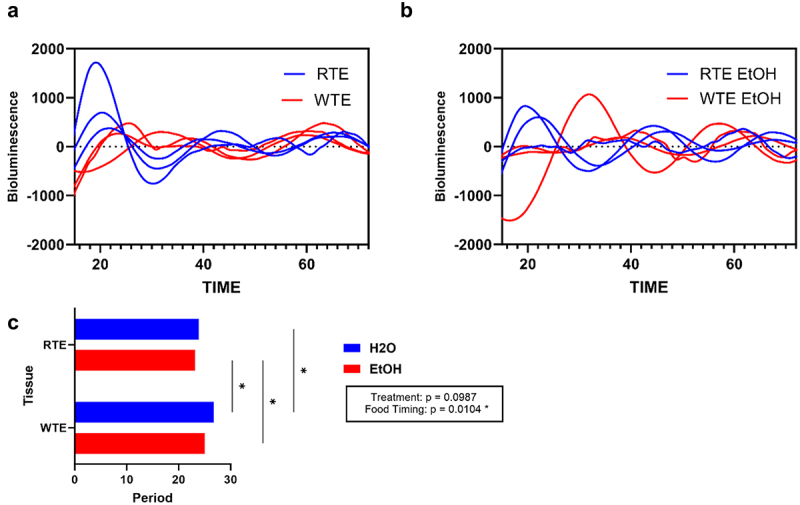
Circadian rhythms of colon tissue. (a) Bioluminescence of PER2:LUC in RTE and WTE colon tissue with control (H_2_O). (b) Bioluminescence of PER2:LUC in RTE and WTE colon tissue with alcohol (EtOH). (c) The period of WTE mice has increasing variability than the periods of RTE in colon tissue. There is a significant difference in the period between RTE and WTE colon tissue. Experiments were performed in triplicate (*n* = 3). Purple bars represent H₂O groups and blue bars represent EtOH groups. Two-way ANOVA (results in box) was conducted, and effects are indicated on each graph when significant: *p < 0.05, **p < 0.01, and ***p < 0.001.

Altered food timing was sufficient to induce significant differences in circadian rhythmicity between RTE and WTE mice (p = 0.0104), increasing variability (shortening and lengthening) in the period of PER:LUC rhythms in the colon (Figure 1) through an increase in the period of PER2:LUC mice rhythms in the colon tissue: RTE H₂O (23.83 ± 0.04 h) and RTE EtOH (23.160 ± 0.73) versus WTE H₂O (26.76 ± 0.95 h) and WTE EtOH (25.01 ± 0.26). This data demonstrates that altered food timing in mice creates more variability (disruption) in their colon tissue circadian clock entrainment.

### Impact of wrong-time eating (WTE) and alcohol on food/liquid consumption and weight in mice

Average weekly food and liquid intake were significantly affected by treatment and sex effects. Males consumed more food, as expected, but consumed less liquid compared to female mice. Body weight was assessed and was significantly affected by alcohol treatment and sex effects. Male mice were more affected by alcohol treatment, resulting in decreased average body weight. Average weekly alcohol intake was measured and there was a significant impact of the food timing paradigm on alcohol consumption (Supplemental Figure S1).

### Impact of wrong-time eating (WTE) on alcohol consumption in mice

The blood alcohol levels were assessed with significantly elevated alcohol effects noted in the right-time eating alcohol-fed group (RTE EtOH), consistent with blood collected following 12 h of food intake and 20% EtOH (v/v) availability. It is significantly increased compared to the H₂O groups. However, there was not a significantly elevated blood alcohol in the wrong-time alcohol-fed (WTE EtOH) group due to a study constraint. The time of sacrifice occurred in the morning although we anticipate this trend to have continued with significantly increased levels if this group was sacrificed at night when the mice are coming off 12 h of food intake and 20% EtOH (v/v) availability (Supplemental Figure S2).

### Impact of wrong-time eating (WTE) and alcohol on intestinal permeability

**Figure 2.**
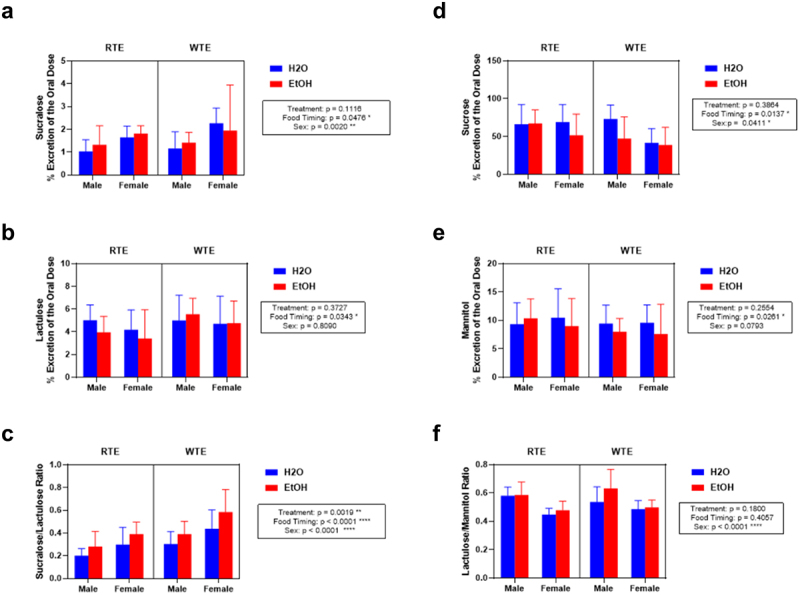
Effect of altered food timing and alcohol consumption on urinary sugar excretion to assess intestinal barrier integrity. (a) Urinary sucralose exhibited a significant effect of food timing and sex but no interaction. (b) urinary lactulose exhibited a significant effect on food timing but no interaction. (c) Sucralose:lactulose ratio was significantly impacted by alcohol treatment, food timing, and sex. (d) urinary sucrose was significantly impacted by food timing and sex, but there was no interaction. (e) Urinary mannitol exhibited a significant effect on food timing and sex but no interaction. (f) Lactulose:mannitol (LM) ratio was significantly impacted by sex, but there was no difference of food timing or alcohol treatment nor was there an interaction. Between *n* = 7–13 mice/treatment group. Three-way ANOVA (results in box) was conducted, and effects are indicated on each graph when significant: *p < 0.05, **p < 0.01, ***p < 0.001, and ****p < 0.0001.

The primary aim of this study was to investigate the impact of peripheral circadian misalignment. We assessed the urinary sugar content excretion of sucralose, lactulose, sucralose:lactulose ratio, sucrose, mannitol, and the lactulose:mannitol (LM) ratio. Apart from the lactulose:mannitol (LM) ratio, there was a significant effect of food timing on the intestinal barrier (Figure 2). Of note, the sucralose:lactulose had significant effects on food timing, alcohol treatment, and sex. For instance, the mean ratio of percent of excretion of sucralose-to-lactulose ratio (total gut permeability) for wrong-time eating mice was significantly higher than right-time eating mice (Figure 2c) and markedly different between male and female mice. Although a ratio of sucralose to lactulose is prone to outliers, this ratio is relevant to gastrointestinal permeability due to the kinetics of the sugar probes. We have previously shown this is a more reliable measure of colonic leakiness than sucralose alone.^30^ This data indicates that wrong-time eating increases intestinal permeability and increases colon vulnerability of the colon to the effects of alcohol consumption.

### Impact of wrong-time eating (WTE) and alcohol on AJC protein expression is decreased in colon tissue

Previous studies of central circadian misalignment resulted in colonic hyperpermeability.^20,21^ The apical junctional complex (AJC) of intestinal epithelial cell proteins contains the tight junction protein zonula occludens protein 1 (ZO-1), occludin, and the adherens junction protein E-cadherin that are key regulators of gut leakiness. We sought to determine whether our altered food timing paradigm and alcohol treatment resulted in changes in the expression of these key AJC proteins that regulate permeability in colon tissue.

**Figure 3.**
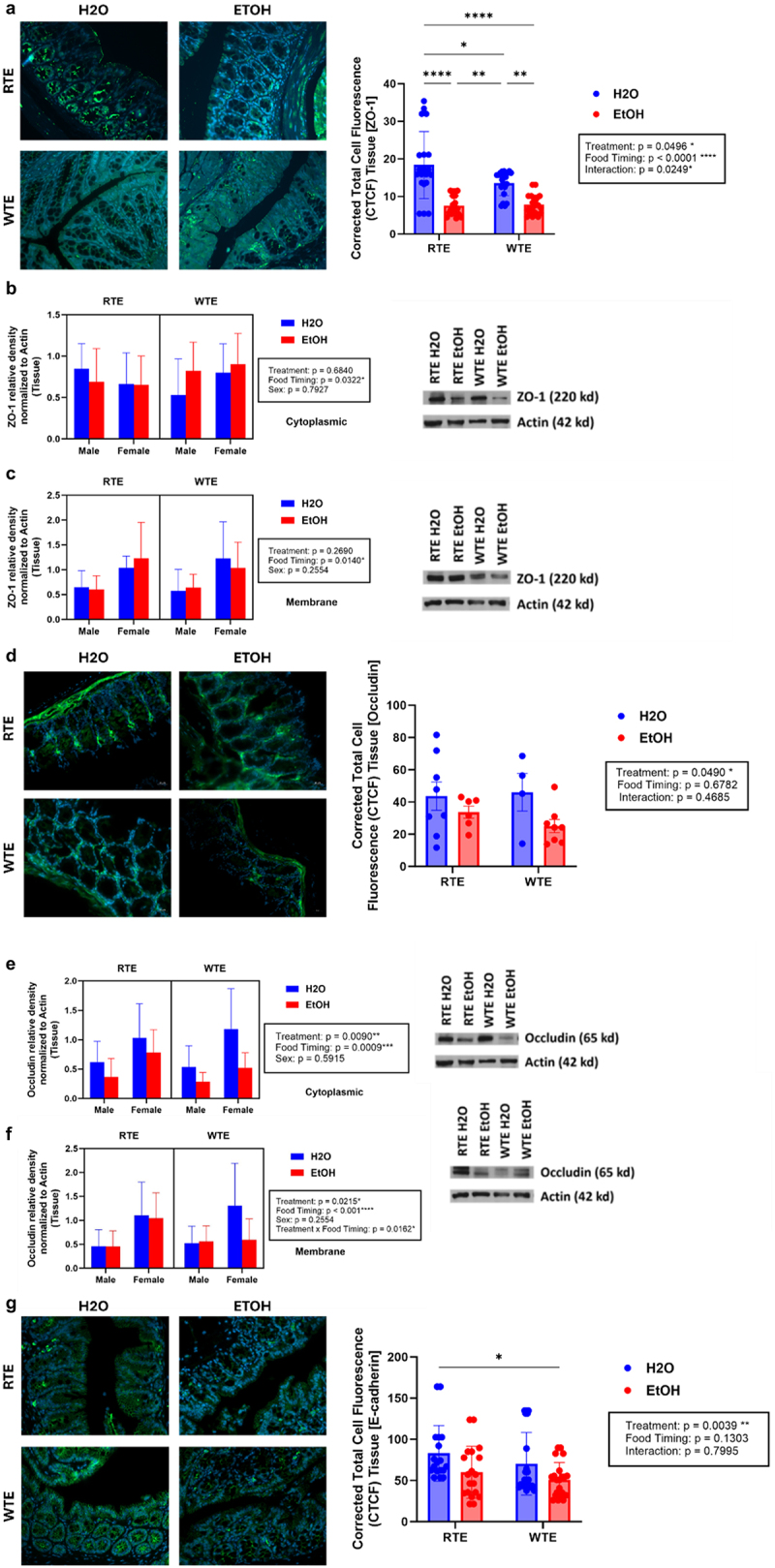
Effect of altered food timing and alcohol consumption on AJC proteins. (a-c) section of colonic tissue labeled with DAPI (blue) and ZO-1 (green). ZO-1 in colon tissue is decreased with altered food timing and alcohol treatment. Between *n* = 19–21 mice/treatment group. (d-f) section of colonic tissue labeled with DAPI (blue) and occludin (green). Occludin in colon tissue decreases with altered food timing and alcohol treatment. (d) Immunofluorescent staining of occludin in colon tissue (left) and cell fluorescent measurement (right), in which alcohol treatment significantly affected occludin expression. (e) Western blot analysis of occludin in its cytoplasmic fraction. Food timing and alcohol treatment effects are significant. (f) Western blot analysis of occludin in its membrane fraction. Food timing, treatment, and their interaction effects are significant. Between *n* = 18–21 mice/treatment group. Three-way ANOVA (results in box) was conducted, and effects are indicated on each graph when significant: *p < 0.05, **p < 0.01, ***p < 0.001, and ****p < 0.0001. (g) Section of colonic tissue labeled with DAPI (blue) and E-cadherin (green). E-cadherin in colon tissue is decreased with alcohol treatment. Alcohol treatment effects were significant. Between *n* = 18–21 mice/treatment group. Two-way ANOVA (results in box) was conducted, and effects are indicated on each graph when significant: *p < 0.05 and **p < 0.01.

First, we assessed ZO-1 through both immunofluorescent staining and western blot analysis (Figure 3a). Total fluorescent measurements were significantly decreased with food timing and alcohol treatment effects. In western blot analysis, food timing significantly decreased the relative density of ZO-1 protein expression (Figure 3b,c). Secondly, we assessed occludin through both immunofluorescent staining and western blot analysis (Figure 3d). Total fluorescent measurements were significantly decreased with alcohol treatment effects. In western blot analysis, food timing and alcohol treatment effects significantly decreased the relative density of occludin protein expression (Figure 3e,f). This data supports that tight junction protein integrity is negatively impacted by peripheral circadian disruption and alcohol consumption. Thirdly, we assessed E-cadherin through immunofluorescent staining (Figure 3g). Total fluorescent measurements of E-cadherin were significantly decreased with alcohol treatment effects. Overall, both altered food timing and alcohol treatment contributed to decreased expression of AJC proteins, mainly in tight junction proteins, that are responsible for maintaining a healthy gut barrier which supports our intestinal permeability data.

### Impact of wrong-time eating (WTE) and alcohol on colonic inflammation.

Colon tissue from mice was assessed for any histological evidence of inflammation or tissue damage (Supplemental Table S1). Male mice from the RTE EtOH group experienced increased scores of histological evidence of inflammation and tissue damage. Overall, there is a sex and alcohol treatment effect (*p = 0.0195*).

### Impact of wrong-time eating (WTE) and alcohol on stool microbiota composition

Intestinal microbiota alterations are observed in alcohol mouse models.^9,31,32^ We first evaluated the impact of altered food timing on stool microbial communities (i.e., RTE H₂O + RTE EtOH vs. WTE H₂O + WTE EtOH). No significant differences in alpha diversity were observed (i.e., variation within each sample) (Table 1); similarly, no significant differences in beta diversity were observed (Table 2) as our food timing model was not enough to induce significant stool microbial changes.

**Table 1.** Alpha-diversity values of mice group comparisons.

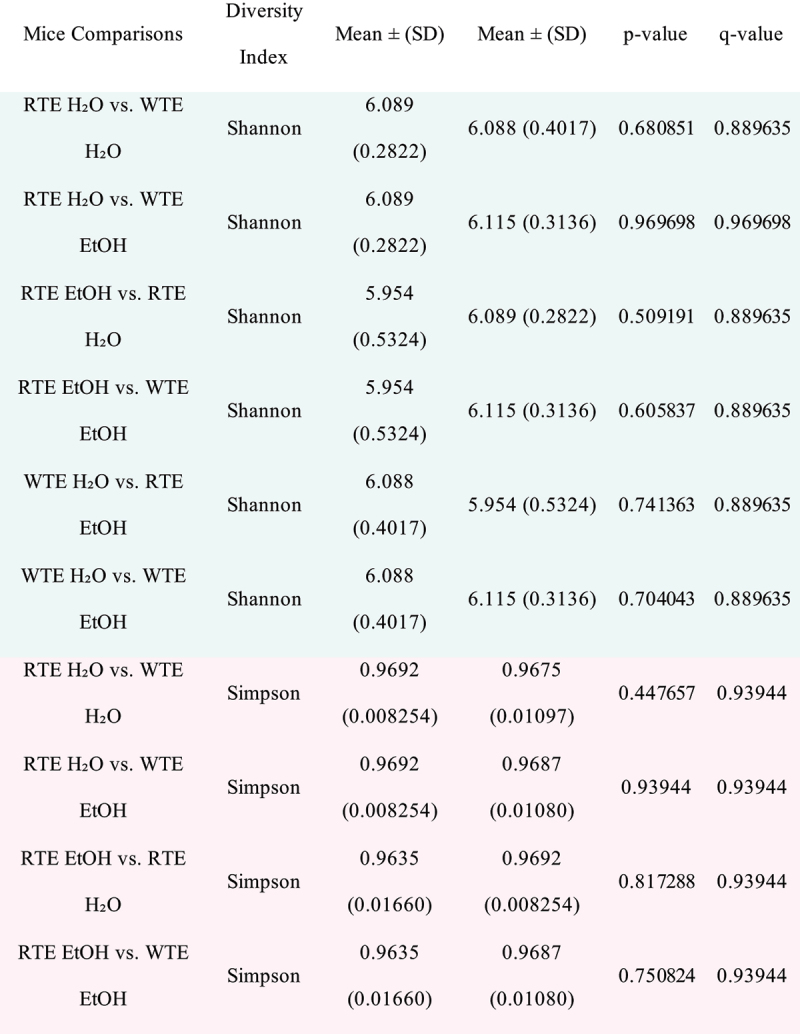	

Alpha diversity values of Shannon’s Index, Simpson’s Index, Observed Features, and Pielou’s Evenness were examined at the feature level. Rarefaction level at 25,000 sequences per sample. The mean index score and standard deviation (SD) are displayed. Total mice sample sizes: RTE H₂O (*n* = 18); RTE EtOH (*n* = 17); WTE H₂O (*n* = 18); WTE EtOH (*n* = 14). The p-values and the adjusted false discovery rate (FDR) q-values are shown.

**Table 2.** Fecal microbial community structure differences between all mice group comparisons as assessed by Permutational Multivariate Analyses of Variance (PERMANOVA).

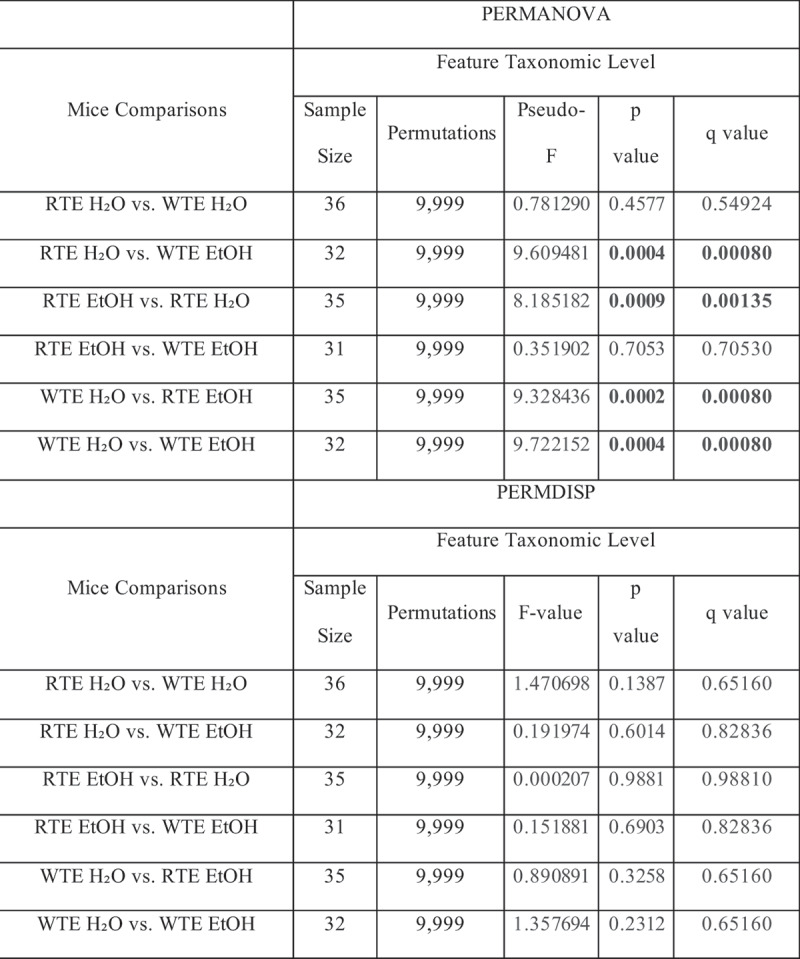	

However, we also evaluated the impact of alcohol treatment effect on stool microbial communities (i.e., RTE H₂O + WTE H₂O vs. RTE EtOH + WTE EtOH). While there were no significant differences in alpha diversity observed (i.e., variation within each sample), the analysis of beta diversity (i.e., variation between samples/ groups) revealed a significant impact of alcohol on the microbiota.

PERMANOVA analysis showed no differences between male and female groups. Subsequent microbiota analysis was run with combined male and female data. Alcohol-fed groups (RTE EtOH and WTE EtOH) had significantly different effects. Alcohol treatment drove the global changes to the microbiota profile seen in our group comparisons (Table 2).

PERMANOVA and PERMDISP results are based on Aitchison matrix distances for the multi-amplicon (V4) sequence data at the feature level. Significant values are based on 9,999 permutations and corrected for multiple testing using the Benjamini–Hochberg method (bolded, q < 0.05). Total mice sample sizes: RTE H₂O (*n* = 18); RTE EtOH (*n* = 17); WTE H₂O (*n* = 18); WTE EtOH (*n* = 14).

We analyzed the distance measure of the samples using Non-metric multidimensional scaling (NMDS) (Supplemental Figure S3). The distance between each group is indicative of each group’s dissimilarity and similarity to each other. The RTE H₂O and WTE H₂O groups are more like each other compared to both the RTE EtOH and WTE EtOH groups.

Phylum and genus taxonomic level differential abundance and compositional analysis of the microbial profiles between mice groups were analyzed with both DESeq2 and centered log-ratio Kruskal–Wallis (CLR-KW). These were compared to Boruta to identify important taxa. CLR-KW identified the same and more taxa compared to DESeq2. Of interest, significant taxa that were identified between H₂O and EtOH groups that were associated with alcohol consumption included: *Clostridium sensu stricto 1*, *Muribaculaeceae*, *Bifidobacterium*, *Turicibacter*, and *Atopobiaceae* (all q < 0.05) (Supplemental Excel sheet S1).

Taken together, the richness (alpha diversity) of the intestinal microbiota was not impacted by either food timing or alcohol treatment. However, the differences between the microbial communities (beta diversity) were driven mainly by alcohol treatment. The identified taxa of significance are associated with alcohol consumption, supporting how alcohol alters the microbial communities in our mouse groups.

### Impact of wrong-time eating (WTE) and alcohol on the microbiota function

**Figure 4.**
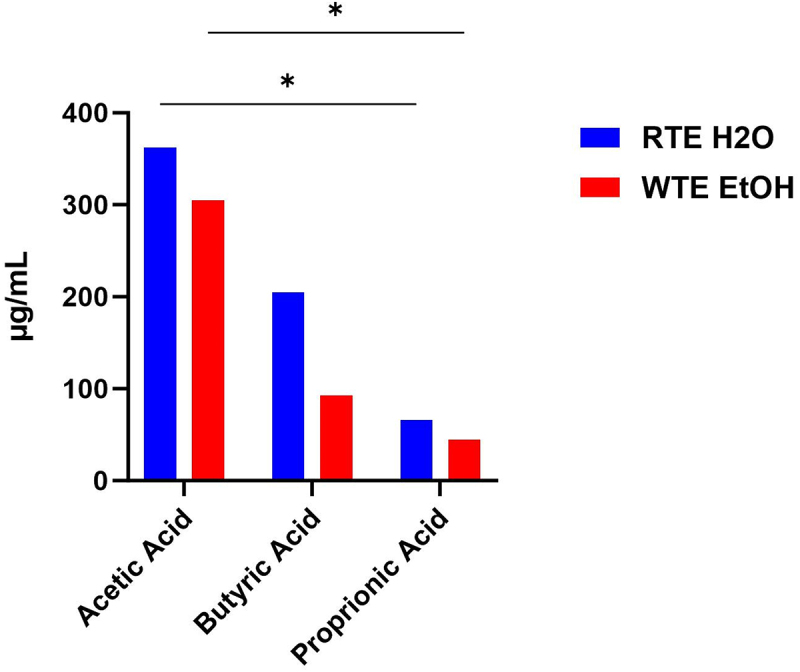
Short-chain fatty acid (SCFA) content in cecal content supernatant of mice. *n* = 4 pooled samples from each respective group, RTE H₂O (blue bars) and WTE EtOH (red bars). Two-way ANOVA was conducted, and the results of multiple comparison tests are indicated on the graph when significant: *p < 0.05.

Lastly, we performed a targeted (short chain fatty acids) metabolomic profile of the cecal content supernatant that was collected from our RTE H₂O and WTE EtOH to have an overall view of their composition (Figure 4). The composition of these cecal supernatants shows a 17% decrease in acetic acid (acetate), a 75% decrease in butyric acid (butyrate), and a 38% decrease in propionic acid (propionate). The largest difference between the RTE H₂O and WTE EtOH was seen in the butyrate levels (75% decrease in the WTE EtOH group). Despite these trends of decreased percentages of the mean SCFA levels across acetic, butyric, and propionic acid, multiple-comparison tests mainly show no differences between the treatment groups. However, we sought to see if this observation is also reflected in the SCFA-producing taxa of these respective groups. Specifically, we anticipate seeing a decrease in butyrate-producing taxa.

**Figure 5.**
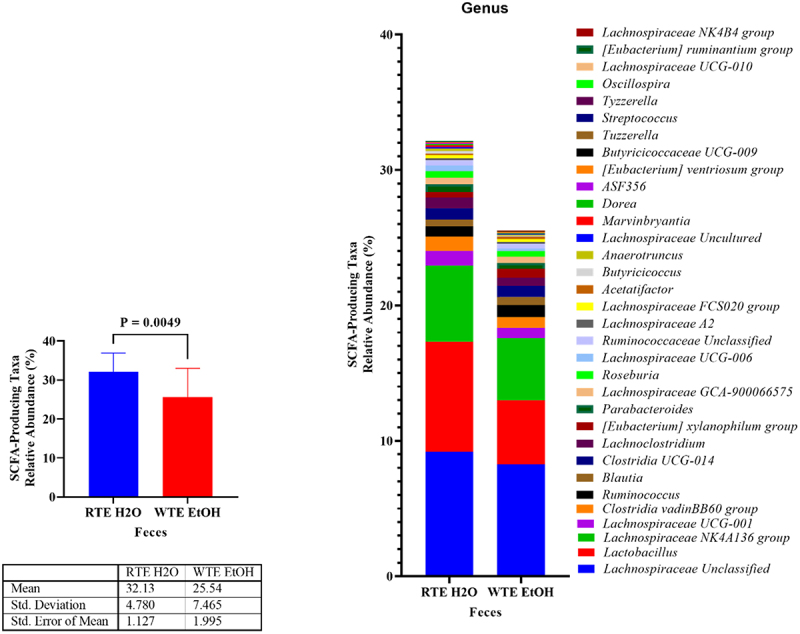
Relative abundance of short-chain fatty acid (SCFA)-producing taxa in the microbiota of RTE H₂O and WTE EtOH mice. Left image: overall percent abundance of SCFA-producing taxa is decreased in the WTE EtOH group (red bar) compared to the RTE H₂O (blue bar). Right image: overall percent abundance of SCFA-producing taxa is decreased in the WTE EtOH group (left) and much of the changes are occurring in *Lachnospiraceae*, an important butyrate producer (right).

Previously, we assessed the phylum and genus taxonomic level differential abundance and compositional analysis of the microbial profiles between mice groups (Supplemental Excel Sheet S1). To complement the metabolomic cecal content data, we sought to identify the relative abundance of SCFA-producing taxa within the two comparison groups (RTE H₂O [blue bars] and WTE EtOH [red bars]). There is a significant difference between the RTE H₂O and WTE EtOH groups with a decrease in the relative abundance of SCFA-producing taxa in the WTE EtOH group (Figure 5). Of the various genera that are decreased in the WTE EtOH group, *Lachnospiraceae*, an important butyrate producer appears most frequently. This also supports the large decrease of butyric acid, butyrate, seen in the WTE EtOH cecal supernatant. When comparing RTE EtOH and WTE EtOH groups for SCFA-producing taxa, there was no difference (p-value = 0.6244) observed (Supplemental Figure S4).

We also assessed the biological pathways present between the RTE H₂O and WTE EtOH cecal content supernatant data using Reactome to predict equivalent human pathways.^33^ A total of 55 pathways were identified (all *p* < 0.05) and a few pathways of interest to note are adherens junctions interactions, G-protein-coupled receptors (GPCR) signaling and binding signaling by GPCR, and γ-aminobutyric acid (GABA) signaling (supplemental Excel sheet S2). These pathways are decreased in the WTE EtOH samples which are implicated in alcohol consumption.^34^

### Relationship between intestinal microbiota and intestinal permeability and barrier integrity outcomes in mice

Significant correlations were noted between sucralose:lactulose ratio and *Turicibacter* (p-value = 0.034269, R-value = 0.394355), Occludin relative density and *Clostridium sensu stricto 1* (p-value = 0.016971, R-value = −0.50322), ZO-1 relative density and *Clostridium sensu stricto 1* (p-value = 0.035408, R-value = −0.42979). These results indicate that *Turicibacter* and *Clostridium sensu stricto 1* may be important mediators of microbiota and epithelial barrier integrity in the context of alcohol consumption and serve as potential targets in future studies (supplemental excel sheet S3).

### Effect of food timing and alcohol treatment on serum and stool inflammatory markers

Food timing and alcohol treatment can induce intestinal inflammation that can subsequently lead to peripheral inflammation in organs throughout the body. We assessed a few inflammatory markers in the serum and stool. First, the pro-inflammatory cytokine IL-6 was examined in the serum for systemic inflammation. While there was no significant difference between groups, there was a slight increase in IL-6 levels seen in the alcohol-treated groups. Second, lipopolysaccharide (LPS) is a component in the outer membrane of gram-negative bacteria, and LPS-binding protein (LBP) is a type 1 acute-phase protein that binds to LPS to facilitate an immune response and is a well-accepted marker of intestinal barrier integrity and endotoxemia. There was a relative increase of serum LBP between food timing in both WTE groups. Third, we assessed calprotectin which is present within neutrophils and throughout the body. Increased stool calprotectin levels are an indication of neutrophil migration into gastrointestinal tissue due to inflammatory processes. Although there was not a significant difference with food timing or alcohol treatment, the levels of calprotectin increased with food timing effects (Supplemental Figure S5).

### Impact of wrong-time eating (WTE) and alcohol on the organoid barrier integrity.

**Figure 6.**
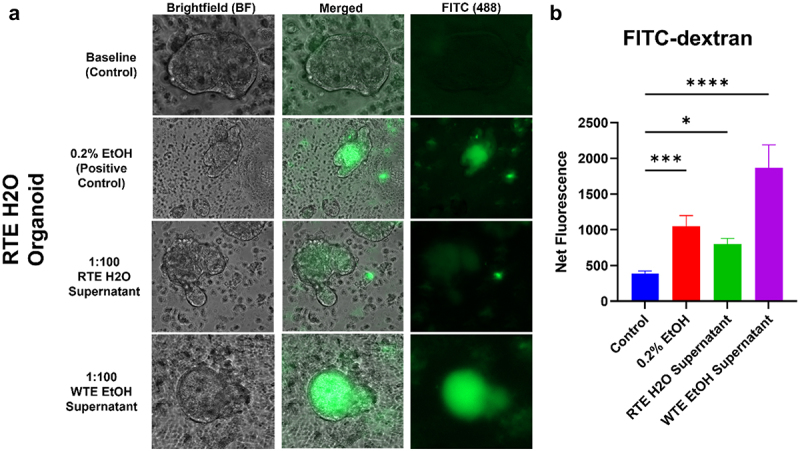
Organoid permeability increases with alcohol and cecal supernatant treatment. (a) FITC-dextran (green) was analyzed at 20x and analyzed for net fluorescence. The presence of green dye inside the lumen of the organoids is indicative of increased paracellular permeability. (b) Net fluorescence levels are significant when comparing the RTE H₂O control (blue bar) to the treatment groups: RTE H₂O cecal supernatant (green bar, *p* = 0.0102), 0.2% EtOH (red bar, *p* = 0.0006), and WTE EtOH cecal supernatant (purple bar, *p*<0.0001). P-values are indicated on each graph when significant: *p < 0.05, **p < 0.01, ***p < 0.001, and ****p < 0.0001.

One key function of the intestinal epithelium is to provide a barrier that separates from the lumen space. The AJC proteins form an intercellular seal that maintains the integrity of the barrier, however this is weakened with alcohol exposure.^18,35,36^ To assess the effects of alcohol and luminal dysbiotic bacteria metabolites from WTE/alcohol-fed mice on paracellular permeability of the organoids, we generated apical-out organoids (physiologically relevant) from control mice (RTE/H₂O-fed mice) and co-cultured the organoids with media (control), 0.2% EtOH (low-dose alcohol that can disrupt intestinal barrier integrity in CaCo-2 cell lines and organoids) or filtered cecal content from RTE H₂O (control mice), or filtered cecal content from WTE EtOH fed mice. Then, the organoids were incubated with FITC-dextran and imaged the organoids using fluorescence confocal microscopy (Figure 6a). The control organoid excluded the FITC-dextran, which was expected for epithelium with intact barrier integrity. For 0.2% EtOH-treated organoids, FITC-dextran diffused into the lumen. For both cecal supernatant treatments, FITC-dextran also diffused into the lumen. Organoid leak induced by the filtered cecal content from WTE/alcohol-fed mice was the highest compared to other organoid treatments. These findings support the idea that the cecal supernatant contains metabolites that impair the intercellular barrier integrity and impact permeability (Figure 6b).

#### AJC protein expression is decreased in colonic organoids.

Previous studies with central circadian misalignment have resulted in colonic hyperpermeability in tissue as well as colonic organoids.^20,21^ The apical junctional complex (AJC) of intestinal epithelial cell proteins contains the tight junction protein zonula occludens protein 1 (ZO-1), occludin, and the adherens junction protein E-cadherin that are key regulators of gut leakiness. The phenotype demonstrated in our *in vivo* mouse model was recapitulated in our *ex vivo* organoid model to compare the colon tissue to the colonic organoids generated from them. We sought to determine whether our altered food timing paradigm and alcohol treatment resulted in changes in the expression of these key AJC proteins that regulate permeability in colonic organoids.

**Figure 7.**
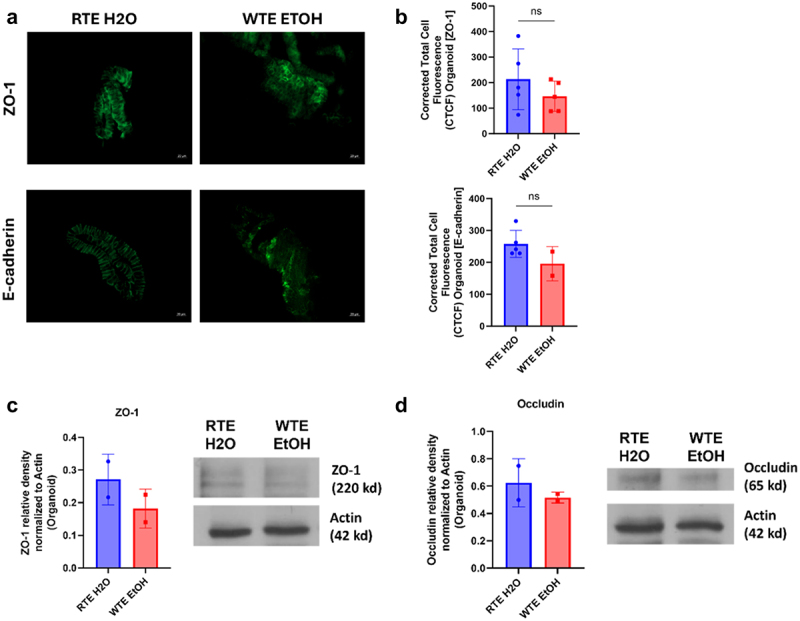
Effect of food timing and alcohol treatment on AJC protein expression in organoids. (a) Immunofluorescent staining of tight junction protein ZO-1 and adherens junction protein E-cadherin (green). (b) No differences in ZO-1 or E-cadherin total fluorescence, however, there is a decrease in the WTE EtOH group. *N* = 5 mice/treatment group. (c) Western blot analysis of ZO-1 relative density. No difference due to low n but a similar trend of decreased ZO-1 expression in the WTE EtOH group. Representative data with *n* = 2 mice/treatment group. (d) Western blot analysis of occludin relative density. No difference due to low n but a similar trend of decreased occludin expression in the WTE EtOH group. The blue bar represents the RTE H₂O group, while the red bar represents the WTE EtOH group. Representative data with *n* = 2 mice/treatment group.

We assessed ZO-1 and E-cadherin through both immunofluorescent staining and western blot analysis (Figure 7). Total fluorescent measurements for ZO-1 and E-cadherin decreased like their tissue counterparts in Figure 6 but were not significant (Figure 7a,b). In western blot analysis, the relative density of ZO-1 and occludin protein expression were decreased like their tissue counterparts in Figure 6 but were not significant (Figure 7c,d). Despite the low *n*, the immunofluorescent staining and western blot data are representative and follow similar trends of decreased expression in the WTE EtOH group.

#### Assessing organoid cellular proliferation

To assess cellular proliferation in the organoids, we stained for the cell proliferation marker Ki-67 (Supplemental Figure 6). Previous organoid studies have shown that alcohol-treated organoids have increased levels of Ki-67.^37^ Alcohol is known to promote cellular proliferation and the migration of intestinal epithelial cells. This data demonstrates an increase in the number of Ki-67 organoids present in the WTE EtOH group.

## Discussion

The key findings of the present study are: 1) wrong-time eating (WTE) induces peripheral circadian disruption in the intestine; 2) peripheral circadian misalignment by WTE decreases alcohol intestinal barrier resiliency; 3) WTE-induced decreased resiliency of intestinal barrier to alcohol injurious effects is mediated through disruption of tight junction proteins – ZO-1 and Occludin; 4) alcohol, not food timing, is the primary trigger for microbiota dysbiosis in our model; 5) WTE and alcohol caused decreased relative abundance of SCFA producers and lower butyrate levels in the colonic lumen; and 6) cecal contents from WTE mice increased sensitivity of organoid barrier to cytokines-induced damage (i.e. recapitulate WTE-induced decreased resiliency of intestinal barrier disruption by alcohol in mice) suggesting that luminal factors like bacterial products mediate WTE effects on intestinal barrier function.

Circadian rhythms in the intestines play a crucial role in regulating various physiological processes including digestion, nutrient absorption, gut motility, and cell proliferation. While these peripheral circadian rhythms synchronize with the central circadian clock, peripheral clocks can behave independently based on certain cues. Feeding and fasting are the main drivers of peripheral circadian rhythms, oscillating with rest and active cycles. However, genetic studies of clock genes—*Cry*, *Per1/2*, *Rev-erbα*, and *Bmal*—demonstrate their effects on intestinal health. For instance, *Bmal* mutants which have a loss of rhythms, negatively influence cellular regeneration in the colon, leading to worsened colitis in murine DSS-colitis models.^38^ In addition to the intestine, perturbations to the circadian clocks have resulted in alterations of the gut microbiota, leading to decreased production of bacterial-derived metabolites like short-chain fatty acids.^24–26^

Previously, we demonstrated how a central circadian misalignment model can promote an increase in colonic permeability and impair barrier integrity.^20–22^ In this study, we wanted to see if peripheral circadian misalignment also promotes an increase in colonic permeability and impaired barrier integrity. To disrupt peripheral (colon) circadian homeostasis, we used an altered food paradigm (WTE) with and without alcohol to assess if we successfully disrupted circadian rhythms in the colon by the WTE paradigm.^9^ Then, we utilized a PER2:LUC gene reporter mouse that expresses a Per2-luciferase fusion protein that allows us to measure both colon tissue and colonic organoid circadian clock rhythmicity.^39,40^ We found that colon tissue had increased variability in the WTE group. Alcohol did not significantly affect circadian rhythmicity suggesting that altered food timing alone is sufficient to affect peripheral circadian rhythms.

To assess the extent of the impact that peripheral circadian misalignment has on the resiliency of the colon, we assessed measures of intestinal permeability, barrier integrity, gut microbiota profiles, as well as the potential effect of the cecal supernatant of these mice on potentially ameliorating and/or exacerbating intestinal outcomes. Altered food timing and alcohol consumption resulted in increased intestinal permeability and exhibited decreased expression of AJC proteins like ZO-1, occludin, and E-cadherin in colon tissue. These AJC proteins are markers of intestinal permeability and are under circadian regulation and this data supports the findings in previous studies.^9,41–43^

However, despite the disruption of intestinal AJC proteins and increased intestinal barrier by WTE and alcohol, we could not find evidence of systemic inflammation (normal serum cytokine) or endotoxemia (normal serum LBP). It is possible that the duration of our study was not adequate to cause long enough disrupted barrier function to cause enough intestinal leak to endotoxin to be identified by changes in serum LBP or serum cytokines. Further studies with a longer duration of WTE and alcohol are required to answer this question.

One possible mechanism for disruption of intestinal barrier integrity by WTE/alcohol is altered microbiota composition (dysbiosis). Indeed, we identified significant taxa that are implicated in alcohol consumption such as *Clostridium sensu stricto 1*, *Muribaculaceae*, *Bifidobacterium*, *Turicibacter*, and *Atopobiaceae*. We also observed a decrease in the relative abundance of putative anti-inflammatory SCFA-producing taxa, like *Lachnospiraceae*, an important butyrate producer.^23,44^ We then used the organoid model to determine whether WTE/alcohol disrupts intestinal barrier integrity more directly and whether luminal dysbiotic bacteria products are responsible for the WTE-induced decreased resiliency of the intestinal barrier to the injurious effect of alcohol. Previous work demonstrated that colon organoids are an excellent model to elucidate the mechanism of the alcohol-induced gut leak or disrupted circadian-induced intestinal epithelial injury because colonic epithelial organoids retain the phenotype of the host.^20,21,42^ Intestinal organoids are self-renewing three-dimensional tissue cultures that are derived from intestinal stem cells and can be used to test specific hypotheses that are not possible *in vivo*. They are advantageous compared to traditional cells used for cell culture (e.g., Caco-2 cells) because the organoids contain various primary cell types present *in vivo*. Indeed, PER2:LUC mice have been a useful model for the characterization of the circadian clock because the insertion of the luciferase reporter in the *Period* gene (a critical component of the molecular circadian clock) allows for easy assessment of Period gene expression across 24 h.^39,40^ Previous studies have studied organoid circadian phenotypes in the small intestine, but few studies have examined colonic organoids. In the current study, we used colonic organoids and found AJC proteins were significantly disrupted in organoids from WTE/alcohol-fed mice. This study complements our previous study which found that central circadian disruption disturbs the circadian regulation of apical junction proteins.^21^ Furthermore, using colonic organoids from control mice (RTE/H_2_O mice), we found that filtered cecal content from WTE alcohol-fed mice induces marked intestinal leak suggesting that colonic lumen in WTE/alcohol-fed mice contain compound(s) that, at least in part, may be responsible for alcohol-induced disruption of intestinal barrier integrity and perhaps WTE-induced decrease resiliency of intestinal barrier to the injurious effect of alcohol.

We found that WTE/alcohol-fed mice have a dysbiotic microbiota community in their stool characterized by a relative abundance of SCFA-producing bacteria. In our prior studies, we also found stool microbiota dysbiosis characterized by a low relative abundance of SCFA producers in central circadian disrupted humans and mice.^12,20–23^ It is noteworthy that multiple studies in rodents and humans have shown the important role of SCFA in maintaining normal intestinal barrier function with low SCFA being associated with disrupted intestinal barrier integrity.^20,23,45^ We therefore measured SCFA levels in the filtered cecal content and found that SCFA, butyrate was significantly decreased in the cecal content of WTE/alcohol-fed mice. Thus, it is highly plausible that low SCFA levels in the cecal content are responsible for intestinal organoid leak induced by the cecal content from WTE/alcohol-fed mice. However, other metabolites/compounds can still be contributing factors to this leak. Further studies including untargeted and targeted metabolomics of the cecal content and direct treatment of organoids with these compounds are required to identify the culprit metabolite(s).

The following are several important limitations of the present study. Firstly, we assessed serum and stool samples at one singular time point in the morning, instead of conducting two (one morning and one nightly collection), at the end of the study. This was a byproduct of the study design which might have impacted the difference between groups in various serum and stool markers (e.g., serum blood alcohol, IL-6, serum LBP, and stool calprotectin). Secondly, while we can recapitulate barrier dysfunction in RTE organoids with cecal contents from WTE mice (demonstrating a loss of function), the assessment of cecal contents from RTE mice on WTE organoids (“gain of function” approach) was not performed. This was in part due to limited WTE organoid availability. Thirdly, the caloric intake was not controlled in our alcohol-in-water model with an isocaloric substitution such as maltodextrin. This decision was based on our prior finding that maltodextrin can also affect the intestinal barrier inflammation readouts and the intestinal microbial community.^46^ Lastly, we performed a global analysis of the cecal content and did not look at specific bacteria or metabolites that should be examined in additional studies. This research paradigm offers valuable insights into how abnormal, late-night eating and alcohol consumption interact with the gut. Although our study focused on mice, these findings may reflect similar trends in humans, such as night shift workers and in groups where individuals tend to stay up late on a regular basis. Understanding the biological effects of these behaviors could help address potential long-term health risks, such as digestive disorders or metabolic dysfunction. Future research could explore targeted interventions, such as rescue treatment with bacterial-derived metabolites like butyrate from non-circadian disrupted individuals or lifestyle adjustments to mitigate the negative consequences of disrupted eating patterns and alcohol intake.

## Conclusion

In summary, our study indicates that: (1) Eating during biological rest time (wrong-time eating [WTE], a model of peripheral circadian misalignment) is sufficient to influence circadian rhythmicity, (2) Circadian misalignment by WTE decreases the resiliency of the colon to the injurious alcohol effect and this effect might be mediated through intestinal microbiota dysbiosis that results in low SCFA in the colonic lumen. Unhealthy alcohol consumption in circadian misaligned hosts is a major contributor to intestinal microbiota dysbiosis and gut leak and identifying the microbial metabolites that mediate gut leak and associated intestinal/systemic inflammation may lead to new microbial and circadian targets to prevent organ damage in patients with alcohol use disorders.

## Materials and methods

### Animal model

All experiments were approved by the Rush University Institutional Animal Care and Use Committee (IACUC). Mice with a Per2 (mPer2Luciferase [mPer2Luc]) knock-in mutation, obtained from Jackson Labs (Bar Harbor, ME), were bred in-house.^39^ A total of 89 mice (51 male and 38 female) were used for experiments, with 12–13 males and 9–10 females. All mice were at least 8-weeks old when the experiments began. The animals were maintained on a 12-h light:12-h dark (12∶12 LD) cycle with lights on at 7 AM and lights off at 7 PM Central Standard Time.^22^ Animals were individually housed in ventilated, light-tight cabinets with 40W GE “Cool White” fluorescent bulbs individually controlled by an electronic timer.^43^

### Food timing paradigm

Mice were placed on a food-restricted paradigm. As mice are nocturnal and consume their food in the dark, food timing in this experiment is defined with either day- (rest-phase= “wrong-time”, 7 AM) or nighttime (active-phase= “right-time”, 7 PM). Mice received access to food for 12 h for 5 consecutive weekdays followed by a 2-day *ad-libitum* access to food to mimic human conditions on the weekend.^9^ Mice were fed Envigo 2018 standard rodent chow (Teklad, Madison, WI) and given either normal drinking water or alcohol in drinking water (described below). This paradigm lasted a total duration of 10 weeks (Supplemental Figure S7). The general condition and health of the mice were monitored by daily observation, weekly body weight, food intake, and liquid intake measurements.^47^

### Alcohol treatment

The control groups received drinking water (H₂O). The mice in alcohol-fed groups were given 20% alcohol v/v treatment in drinking water.^48^ The 20% alcohol was only provided during the mice’s food intake periods, so it was available *ad-libitum* during the weekends when the mice had unrestricted access to both food and liquid.^49^ Rodents reportedly metabolize alcohol much more rapidly than humans, at around 5.5 times the human rate.^50^ To achieve blood alcohol levels (BAL) in mice that are pharmacologically relevant and comparable to BAL in alcohol-consuming human (~1.0 g/L), a 20% alcohol dose in drinking water was required.^51^ To acclimate the mice to alcohol consumption, a 14-day ramp-up was used, starting at 3% on days 1–3, increasing to 5% on days 4–7, 10% on days 8–10, 15% on days 11–14, and then reaching the target treatment of 20% on day 15. The volume was replaced as needed, with a maximum fill of 30 mL, and daily intake of both water and alcohol was measured. After 10 weeks, spontaneously voided stool samples were collected with sterile forceps to assess the intestinal microbial communities, followed by a urine test for intestinal barrier integrity. Finally, at the end of the study, the mice were euthanized via guillotine. Then, trunk blood and tissue samples were collected (Supplemental Figure S8).

### Intestinal barrier (permeability) testing

The mice were fasted overnight prior to the urine permeability test, which is performed the next morning.^22,30^ A 200 µl solution containing lactulose (3.2 mg), sucrose (0.45 mg), sucralose (0.45 mg), and mannitol (0.9 mg) is administered via oral gavage, followed by a subcutaneous injection of 2 mL of 0.9% saline to promote urine production. The mice were then placed into a metabolic chamber with water available *ad-libitum* for 5 h, after which the urine produced was collected and the total volume was recorded. Urine samples were stored at −80°C until further experimental analysis. This 5-h timeframe allows for intestinal transit, enabling assessment of barrier integrity across the entire intestine, including the colon. Intestinal barrier integrity is determined by measuring urinary concentrations of the administered sugars using gas chromatography, which enables calculation of the percentage of the oral dose that was exerted in the urine over 5 h. Then, intestinal permeability is assessed at the end of the 10-week study period.^30^

### Intestinal microbiota analysis

#### Stool collection

To collect stool samples for microbiota analysis, individual mice were housed in a bedding-free cage for 12 h. After this 12-h period, the spontaneously produced stool pellets were gathered using sanitized forceps and stored at −80°C until further experimental analysis.

#### DNA extraction

The total genomic DNA was extracted from end-of-study stool pellets using the FastDNA SPIN Kit. The extracted DNA was then quantified using fluorometric quantitation on a Qubit 3.0 instrument (Life Technologies, Grand Island, NY, USA). To decrease run effects, all samples were extracted together using the same DNA extraction kit, and library preparation was conducted concurrently in 96-well plates.

The variable region 4 (V4) of microbial 16S ribosomal RNA (rRNA) genes was targeted for PCR amplification using the modified Earth Microbiome Project primers 515F/806 R (515F: GTGTGYCAGCMGCCGCGGTAA; 806 R: CCGGACTACNVGGGTWTCTAAT). The PCR method, as previously described done in a two-stage fashion, was used to prepare the amplicons for high-throughput sequencing.^52,53^ The amplicon libraries were sequenced on an Illumina MiniSeq, utilizing a V2 kit and generating paired-end (150) base reads, at Rush University Medical Center utilizing the Rush Genomics and Microbiome Core Facility.

### 16S rRNA V4 sequencing analysis

PEAR (paired-end read merger) (v0.9.11) software package (Dalhousie University, Halifax, Nova Scotia, Canada) was used to analyze merged raw sequences.^54^ A DADA2 algorithm from the QIIME2 (v 2020.8.0) workflow was utilized to remove sequences shorter than 240 bases, with denoising, were then removed before the merged were processed.^55,56^ The resulting amplicon sequence variants (ASVs) were used for all subsequent analyses. Each ASV was allotted a taxonomy using an unbiased Bayes classifier and the SILVA 138 99% database reference for OTUs.^57,58^ In total, 4,229,677 sequencing clusters were generated, with an average depth of 44,059 sequences per sample (median = 41,906; min = 1; max = 113,563). Three reagent contaminant ASVs (*Rhodococcus, Allorhizobium-Neorhizobium-Pararhizobium-Rhizobium*, and *Pseudomonas*) were identified and removed using the decontam algorithm based on their prevalence in the reagent negative blank controls (*n* = 4).^59^ Unassigned and host-associated taxa, such as mitochondrial ASVs, chloroplast, and eukaryote were also removed from the datasets prior to statistical analyses.^60^ After analysis, the raw sequence data were deposited in the NCBI Sequence Read Archive under BioProject PRJNA881333.

### Blood collection and tissue harvest

At the end of the study, the mice were euthanized via guillotine. Blood was collected by trunk collection, then centrifuged at 2,000 RPM for 35 min at 22°C. The serum was collected and stored at −80°C until experimental analysis. Murine tissue samples were collected: taking sections of the liver and the large intestine. The large intestine was flushed clean with cold phosphate-buffered saline (PBS) at least three times to remove excess debris. The tissue segments were snap-frozen and stored at −80°C, or formalin-fixed and paraffin-embedded (FFPE), as previously described.^42,61^

### Per2-luciferase bioluminescence

Rhythmic expression of the clock gene PER2 (PER2 luciferase, PER2:LUC) persists in these colonic tissues and organoids, allowing constant monitoring of the robustness of these oscillations.^40^ Freshly collected mouse colonic tissue was immediately transferred onto Matrigel-coated 35 mm dishes to minimize tissue movement in the media-filled dishes. Floating colonic organoids were also assessed in these dishes separately. The Kronos Dio apparatus (AB-2500, Atto Co., Tokyo, Japan) quantified bioluminescence levels of the PER-2 luciferase protein (E1602, Promega) in luciferin-containing media. Measurements were taken for 1 min every 15 min, at a controlled temperature of 37°C and 5% CO2, for a minimum of 5 days.

### Mouse organoid preparation and culture

The mouse intestinal epithelial organoid cultures from the colon were prepared using a previously described method.^42,62–64^ Briefly, the colon was washed with ice-cold PBS lacking Ca++ and Mg++ (Thermo Fisher Scientific, #100-10-023) to remove any loose debris. The samples were cut longitudinally, and excess fat was gently scraped off. After two PBS washes, the samples were cut into small pieces and transferred into a 50 mL conical tube. The tissue was then washed five times with PBS, transferred to a separate 50 mL conical with 2.5 mm EDTA chelating buffer, and incubated on a shaker at 4°C for 1 h. The supernatant was removed, and the tissue pieces were washed in PBS, discarding the first fraction. Three mL of PBS was added, and the crypts were gently resuspended and filtered through a 70 μm cell strainer into a fresh conical tube. This washing and filtering was repeated three times. The crypt suspension was centrifuged at 300 g for 5 min after adding 10% FBS. The supernatant was discarded, and the cell pellet was resuspended in 15 mL of organoid media minus growth factors. Base organoid media consisted of: Advanced DMEM/F-12 (Thermo Fisher Scientific), Glutamax (Thermo Fisher Scientific), Penicillin/Streptomycin (Sigma Aldrich), HEPES (Sigma Aldrich), B27-supplement (Thermo Fisher Scientific), N2-supplement (Thermo Fisher Scientific), and N-Acetylcysteine (Sigma Aldrich).

The cell suspension was then centrifuged at 150 g for 3 min. Then, this washing/centrifugation step was repeated twice. After the final centrifugation, the crypts were counted, resuspended in cold Matrigel (60 μL per well), and plated on a warm 24-well culture plate. Then, the plate was incubated at 37°C for 30 min to allow the Matrigel to polymerize before adding 500 μL of organoid medium with the growth factors to each well. Growth factors for colonic organoids included: recombinant mouse EGF (Sigma Aldrich), recombinant mouse Noggin (Sigma Aldrich), recombinant human R spondin 1 (PeproTech), recombinant mouse WNT-3A (PeproTech), A-83-01 (Tocris Bioscience), SB202190 (Sigma Aldrich), Y-27632 (Sigma Aldrich), and nicotinamide (Sigma Aldrich). The organoids were incubated at 37°C/5% CO2. Organoids were observed daily to ensure proper organoid health and robustness. Then, the organoids were passaged at least three times (roughly 3 weeks from the initial preparation) before any experimentation was performed.

### Apical out intestinal organoids

We generated apical out organoids from these initial organoids using a previously described method.^65^ Organoid growth was assessed under the microscope daily. To ensure proper health and robustness, the organoids were passaged at least three times (approximately 3 weeks from the initial preparation) before any experimentation was performed.

### Cecal content supernatant

The cecal content supernatant sample preparations were adapted from previously published methods.^66^ The cecal contents were pooled together (*n* = 4) for each group, weighed on ice, and 500 μL of cold PBS was added 100 mg cecal content. The sample tubes were then vortexed for 5 min and centrifuged at 13,000 g for 15 min at 4°C. The resulting supernatant was filtered through a 0.22 μM membrane with a 1 ml syringe before the sample was stored at −80°C until experimental use.

#### Sample preparation

The cecal content supernatant samples were prepared with the automated MicroLab STAR® system from Hamilton Company from previously described methods from Metabolon.^67^ Several recovery standards were added prior to the first extraction step in for quality control purposes. To extract chemically diverse metabolites while removing proteins, the cecal content supernatant samples were precipitated with methanol under vigorous shaking for 2 min, using a Glen Mills GenoGrinder 2000, followed by centrifugation. This helped dissociate small molecules bound to or trapped in the precipitated protein matrix. The resulting sample extract was divided into five fractions: two for analysis by positive ion mode electrospray ionization (ESI) reverse phase (RP)/ultra-performance liquid chromatography (UPLC)-tandem mass spectrometry (MS/MS), one for negative ion mode ESI RP/UPLC-MS/MS, one for negative ion mode ESI hydrophilic interaction liquid chromatography (HILIC)/UPLC-MS/MS, and one sample was reserved as a backup. Finally, organic solvent was removed from the samples then stored overnight under nitrogen before preparation for analysis.

#### Ultrahigh performance liquid chromatography-tandem mass spectroscopy (UPLC-MS/MS)

The analysis utilized a Waters ACQUITY UPLC system coupled with a Thermo Scientific Q-Exactive high resolution/accurate mass spectrometer using previously described methods.^68^ The mass spectrometer was equipped with a heated electrospray ionization (HESI-II) source and Orbitrap mass analyzer, operated at 35,000 mass resolution. The cecum content supernatant sample extract was dried and reconstituted in solvents compatible with analytical standards at fixed concentrations to ensure consistent injection and chromatographic. Four different ionization methods were used to analyze the samples. One aliquot was analyzed using acidic positive ion conditions using a C18 column (Waters UPLC BEH C18–2.1×100 mm, 1.7 µm) and a gradient of water and methanol, containing 0.1% formic acid (FA) and 0.05% perfluoropentanoic acid (PFPA). This method was optimized for more hydrophilic compounds. The second aliquot was also analyzed using acidic positive ion conditions using the same C18 column, but with a gradient of acetonitrile, methanol, water, 0.05% PFPA, and 0.01% FA. This method was optimized for more hydrophobic compounds and operated at a higher overall organic content. A third aliquot was analyzed under basic negative ion optimized conditions using a separate dedicated C18 column. Utilizing methanol and water with 6.5 mm ammonium bicarbonate at pH 8 the basic extracts were gradient eluted. The final, fourth aliquot was determined by negative ionization by elution with a HILIC column (Waters UPLC BEH Amide 2.1 × 150 mm, 1.7 µm). About 10 mm ammonium formate at pH 10.8 with a gradient of water and acetonitrile was utilized. A scan range across 70–1000 m/z alternated between MS and data-reliant MS scans using dynamic prohibition was performed.

### Fluorescein isothiocyanate (FITC)-dextran

To assess epithelial barrier integrity, apical-out organoids were treated for 3 h with either control media, 0.2% EtOH, or a 1:100 dilution of cecal content supernatant (RTE H₂O or WTE EtOH) with control media. The organoids were then pelleted, and washed in PBS (4°C, 5 min, 5,000 RPM) before being incubated for 30 min in a solution (2 mg/ml) of 4 kDa fluorescein isothiocyanate (FITC)-dextran (Sigma, FD4-1 G) in a 37° incubator.^65^ Organoids are pelleted and washed (4°C, 5 min, 5,000 RPM) in a live cell imaging solution (Thermo Fisher, #A14291DJ). Then, the organoids were placed in 8-well chamber slides (Nunc, Rochester, NY) and subsequently imaged using a Zeiss LSM 700 confocal microscope. All images were taken at × 20 magnification with 20–25 enteroids per group. Then, all experiments were performed in triplicate. Data are reported as mean ± standard error of the mean (SEM).

### Immunofluorescent staining of colon tissue and organoids

#### Colon tissue

The colon tissue immunofluorescence (IF) staining was performed on formalin-fixed and paraffin-embedded mouse tissues cut into 5 μm sections. After deparaffinization and rehydration antigen retrieval was carried out using 1X Dako antigen retrieval solution (Agilent # S1699). The slides were then stained with adherens and tight junction protein antibodies for Occludin (mouse, Invitrogen #33–1500), ZO-1 (rabbit, Invitrogen #40–2200), and E-cadherin (mouse, Cell Signaling #14472S). Tissue staining data was collected from *n* = 9–10 mice per group, with images taken from at least 10 stained tissues. The relative expression of each marker was quantified using ImageJ software (ImageJ, U. S. National Institutes of Health, Bethesda, MD) and representative images were selected. All staining was evaluated by two blinded independent observers using a Zeiss LSM 700 confocal microscope. All images were taken at × 40 magnification. The immunofluorescence of each antibody was quantified by calculating the mean difference between background and overall fluorescence of each region. The fluorescence values for each image and section were then averaged to determine the immunofluorescence value for each mouse, using GraphPad Prism (v9.1) software (GraphPad Software, La Jolla, CA).

#### Organoids

The organoid immunofluorescent staining was completed on 8-well chamber slides. The staining occurred after three weeks or longer after colon isolation and organoid generation, allowing optimal colonic organoid growth. The organoids in Matrigel were washed three times with cold PBS after the media was removed. Then, 500 μL of Corning Cell Recovery Solution was added to each well, and the Matrigel suspension was moved to a 15 mL conical tube. An additional 500 μL of Cell Recovery Solution was then added to wash the well. To fully dissolve the Matrigel, the samples were kept on ice for 1 h, with regular inversion. The cells were then spun at 300 g for 5 min. The cells were washed twice more with ice-cold PBS after the supernatant was removed.

The organoids were fixed with 4% paraformaldehyde (PFA) on chamber slides for 1 h at room temperature, and then permeabilized with 1% Triton-X100. The samples were blocked with a solution of 1% bovine serum albumin (BSA), 3% goat serum, and 0.2% Triton X-100 for 1 h. The primary staining for the organoids was performed for 24 h at room temperature in a humidified chamber. On the second day, the slides were washed and incubated for 2 h with their relevant secondary antibodies conjugated to Alexa Fluor 488. The samples were further stained for DAPI and mounted using Fluoromount aqueous mounting medium (Sigma Aldrich), after washing.

A Zeiss LSM 700 confocal microscope was used to image the tissue and organoid slides. The specific antibodies for staining are detailed in Supplemental Table S2. All organoid staining data are from *n* = 1 mice with images from at least 10 stained organoids. Section areas were used to determine the relative expression of each marker using ImageJ software and to pick the representative images for analysis. All staining was evaluated by two blinded independent observers using a Zeiss LSM 700 confocal microscope. All images were taken at × 40 magnification. The immunofluorescence of each antibody was quantified by calculating the mean difference between the background and overall fluorescence of each region. The fluorescence values for each image and section were then averaged to determine the immunofluorescence value for each mouse, using GraphPad Prism (v9.1) software.

### Western blot analysis

Tissue samples were immediately frozen in liquid nitrogen and stored at −80°C until used for protein extraction and concentration prior to western blot analysis. To ensure organoid growth and robustness for experimentation, the organoid protein was collected at least 21 days (approximately 3 weeks) after colon isolation and organoid generation. The commercially available kit, NE-PER Nuclear and Cytoplasmic Extraction Reagents (Thermo Scientific 78,833), was used to obtain cellular material from both colon tissue and organoid samples.

2-mercaptoethanol (Bio-Rad) in a laemmli sample buffer with was used to prep the samples. For 2 h each gel was electrophoresed at 100 V with 20 μg of protein per lane. A 4%/7.5% stacking acrylamide Tris gel was used. As previously described, the membrane transference, blocking, and antibody incubation of both primary and secondary were performed.^20,21^ Autoradiography film (HyBlot CL, Denville Scientific, Metuchen, NJ) with chemiluminescent substrate (ECL, GE Healthcare) was then placed on the membrane, and protein imaging was attained. Image J software was used to examine optical density by densitometric analysis. The specific antibodies are detailed in Supplemental Table S3 (below).

### Hematoxylin and eosin staining

To assess histologic changes within the colonic tissue, 4- to 5-µm thick sections of fixed colon tissue samples were stained with hematoxylin and eosin, as previously described.^61,69^ A gastrointestinal pathologist blinded to the sample identities, scored the tissues across seven categories to measure colon inflammation, tissue injury, and repair: inflammatory cells (0–4), goblet cells (0–4), mucosa thickening (0–4), submucosa cell infiltration (0–4), destruction of architecture (0–4), ulcers (0–1), and crypt abscess (0–1).^61^

### Lipopolysaccharide-binding protein assay

Lipopolysaccharide (LPS) is a component found in the outer membrane of Gram-negative bacteria. LPS-binding protein (LBP) is a type 1 acute-phase protein that binds to LPS, facilitating an immune response. LBP is a well-accepted marker of intestinal barrier integrity and endotoxemia. Serum collected at the time of euthanasia was stored at −80°C until experimental analysis to measure LBP levels using an LBP ELISA kit (Hycult Biotech, HK205), following the manufacturer’s instructions.

### Serum interleukin-6 analysis

Serum levels of the cytokine interleukin-6 (IL-6) were measured using a Meso Scale V-PLEX Pro-inflammatory Mouse Kit (Cat. #K152QXG–1, Meso Scale Diagnostics, Rockville, MD). All samples were analyzed in duplicate on a QuickPlex SQ120 instrument (Meso Scale Diagnostics), following the manufacturer’s protocol.

### Stool calprotectin

At the end of the study, mouse fecal samples were collected and stored at −80°C until experimental analysis. The fecal samples were subjected to only a single freeze-thaw cycle to maintain stool integrity. The calprotectin levels were measured using an ELISA kit (Cat. #KR6936, Eagle Biosciences, Amherst, NH).

### Blood alcohol levels

Blood samples (25 µl each) were removed from storage at −80°C and thawed at 4°C for 15 min. To each sample, a standard (0.1%, or 0.804 mg/ml of n-propanol [nPrOH]; 5 μL) was then added to each sample. Each sample was then transferred and mixed to a 2 mL GC vial by pipette, which was capped right away. The sealed samples were placed on the GC autosampler and allowed to equilibrate at room temperature (22–23°C) for a minimum of 15 min prior to injection. The 25 μl headspace vapor from each sample was injected into a Trace 1310 GC coupled to a Thermo ISQ-LT MS, with a split ratio of 10:1 using a gastight 100 μl syringe. The GC inlet was held at 250°C. A 30 m DB-WAX UI column (0.25 mm ID, and 0.25 μm film thickness) was used to achieve optimal separation. The oven temperature was kept at 40°C for 4 min, and then ramped at 40°C/min to 120°C and held for 1 min. Helium carrier gas flow was maintained at 1.2 mL/min. 250°C was used for both transfer line and ion source. The SIM mode was used to scan ions, at m/z 31 and 45 (EtOH), m/z 31 and 42 (nPrOH), and m/z 59 and 43 (iPrOH [IPA]).

### Statistical analysis

#### Tissue and organoid analyses

Data are reported as mean ± standard error of the mean (SEM). A two-way analysis of variance (ANOVA) was used to evaluate the main effects of food timing (RTE vs. WTE), treatment (H₂O vs. EtOH), and their interaction. Planned a priori comparisons between groups were conducted using a Tukey test to control the type I error.^70^ A separate two-way ANOVA assessed sex examining the main effects of treatment (H₂O vs. EtOH), food timing (RTE vs. WTE), and their interaction. Statistical significance was set at *p* < 0.05. All analyses were performed using GraphPad Prism (v9.1) software.

Spearman’s correlation was used to evaluate the relationships between the relative abundances of specific taxa (species) and phenotype, identifying potential targets for future investigation. A significant threshold was set at *p* < 0.05 and *R* > 0.30.^71^ This approach has been applied in prior studies to uncover associations between bacteria and alcohol consumption.

#### Microbiota analysis

Analyses of alpha- and beta-diversity were used to compare the stool microbial community structure between groups (e.g., EtOH vs. H₂O; WTE vs. RTE) with feature (ASV) counts. Alpha-diversity metrics (Shannon Index, Simpson’s Index, Observed Features (number of taxa), and Pielou’s Evenness (relative abundance of those taxa) were calculated on rarefied datasets of 25,000 sequences/sample, with significance level set at *p* < 0.05. These analyses were conducted using GraphPad Prism (v9.1) software.

Then, Permutation Multivariate Analysis of Variance (PERMANOVA) and Permutational Analysis of Multivariate Dispersions (PERMDISP) were used to compare microbial community structure, with significance determined using 9,999 permutations and corrected using the Benjamini–Hochberg method (q < 0.05).^72,73^ Principal coordinates analysis (PCoA) based on a Bray-Curtis dissimilarity distance matrix was used to visualize baseline microbial community structure of group comparisons (RTE vs. WTE and H₂O vs. EtOH) within the software package QIIME2.^56^ Non-metric multidimensional scaling (NMDS) plots were also generated to visualize bacterial species community data for these group comparisons (RTE H₂O, RTE EtOH, WTE H₂O, and WTE EtOH). Each sample was connected to a centroid representing the mean value of the group.

Boruta feature selection was performed in R.^74^ Both Centered Log-Ratio Kruskal Wallis (CLR-KW) and DESeq2 testing were performed to identify significantly differentially abundant features between mice groups, with Benjamini–Hochberg method correction (q < 0.05).^75–77^ Features with an average relative abundance below 0.1% were removed from the analysis. Finally, individual taxa percent mean relative abundances (˃1%) ± standard deviations (SD) were calculated and depicted as stacked histograms.

#### Serum gas chromatography–Mass spectrometry analysis

The GC/MS data was analyzed using Chromeleon software. This allowed for visual inspection of the retention time and peak area integration for each target analyte (EtOH, nPrOH, and iPrOH). The peak areas for EtOH and nPrOH were then extracted from the data for each sample. Absolute quantitation (in mg/mL and percentage) was calculated using the linear regression equation generated from the calibration curve for each compound. Then, peaks were quantified using the area-under-the-curve method.

#### Cecal content pathway analysis

The sample data was analyzed with Reactome software.^78^ Mouse data identifiers were converted to their human equivalents before analysis was performed. Reactome curates human pathways and infers their existence in other species using orthology information from the ENSEMBL Compara database.^33^

#### Circadian rhythmicity

Analysis of variance (ANOVA) was used to detect the differences in circadian period between right-time and wrong-time mouse groups. All analyses set significance levels at *p* < 0.05. The statistics were performed using GraphPad Prism (v9.1), SPSS (v26) (IBM Incorporation), and circular data was measured by Oriana (v4). To analyze the luciferase time-series data, Biodare 2 was used. Periodogram was performed using the Fast Fourier Transformation Nonlinear Least Square algorithm (FFT-NLLS) to measure period, acrophase, and amplitude.^79^ FFT-NLLS is a variation of the cosinor method and is less sensitive to missing or noisy data. The cosinor fit equation is: $Y\left(t\right)=M+A\cos\left(2\pi t/\tau +\phi \right)$ where M is the MESOR (Midline Statistic of Rhythm, a rhythm-adjusted mean), A is the amplitude (a measure of half the extent of the variation within the cycle), ϕ is the acrophase (a measure of the time of overall highest value), and τ is the period.^80,81^ The circular variance is calculated as [V = 1-r], where *r* = length of the mean vector, and V = Circular variance. Circular variance has been found to be an important marker in genetic models of circadian disruption.^82^ All circular statistics were calculated using Oriana (v4).

## Supplementary Material

Supporting_Documents_for_Food_Timing_Alcohol_Manuscript.docx

## Data Availability

Sequencing reads generated in this study have been deposited in the National Center for Biotechnology Information (NCBI) BioProject database under accession numbers: PRJNA881333. For additional questions regarding data, analytical methods, and study materials, please reach out to the corresponding author(s).

## Synopsis

Peripheral circadian misalignment, modeled through altered food timing, primes the intestinal barrier to further injury by an injurious agent such as alcohol. This phenotype increases intestinal permeability, impairs barrier integrity, and promotes changes in the gut microbiota.

## Abbreviations

Amplicon sequence variants: ASV
Analysis of variance: ANOVA
Apical junctional complex: AJC
Aryl-hydrocarbon receptor nuclear translocator-like 1: ARNT1 also called BMAL1/2
Blood alcohol levels: BAL
Bovine serum albumin: BSA
Carbonic anhydrase 2: CA2
Centered Log-Ratio Kruskal-Wallis: CLR-KW
Circadian locomotor output cycles kaput: CLOCK
Circadian rhythm disruption: CRD
Circadian rhythms: CR
Cryptochrome: Cry
Cytokeratin 20: Krt-20
Ethanol: EtOH
Fast fourier transformation nonlinear least squares: FFT-NLLS
Fluorescein isothiocyanate: FITC-dextran
Formalin-fixed and paraffin-embedded: FFPE
Gastrointestinal tract: GIT
Genomics and microbiome core facility: GMCF
Immunofluorescence: IF
Immunoprecipitation: IP
Inflammatory bowel disease: IBD
Institutional animal care and use committee: IACUC
Interleukin-6: IL-6
Intestinal epithelial cell: IEC
Irritable bowel disease: IBS
Light:dark: LD
Lipopolysaccharide: LPS
LPS-binding protein: LBP
Midline statistic of rhythm: MESOR
Non-metric multidimensional scaling: NMDS
Paraformaldehyde: PFA
PERIOD2 luciferase: PER2:Luc
Permutation multivariate analysis of variance: PERMANOVA
Permutational analysis of multivariate dispersions: PERMDISP
Right-time eating: RTE
Short chain fatty acid: SCFA
Wrong-time eating: WTE

## References

[1] Aschoff J. Exogenous and endogenous components in circadian rhythms. Cold Spring Harbor Symposia Quant Biol. 1960;25:11–27. doi: 10.1101/sqb.1960.025.01.004. 13684695

[2] Takahashi JS, Hong HK, Ko CH, McDearmon EL. The genetics of mammalian circadian order and disorder: implications for physiology and disease. Nat Rev Genet. 2008;9(10):764–775. doi: 10.1038/nrg2430. 18802415 PMC3758473

[3] Green CB, Takahashi JS, Bass J. The meter of metabolism. Cell. 2008;134(5):728–742. doi: 10.1016/j.cell.2008.08.022. 18775307 PMC3760165

[4] Schernhammer ES, Laden F, Speizer FE, Willett WC, Hunter DJ, Kawachi I, Fuchs CS, Colditz GA. Night-shift work and risk of colorectal cancer in the nurses’ health study. J Natl Cancer Inst. 2003;95(11):825–828. doi: 10.1093/jnci/95.11.825. 12783938

[5] Fonken LK, Workman JL, Walton JC, Weil ZM, Morris JS, Haim A, Nelson RJ. Light at night increases body mass by shifting the time of food intake. Proc Natl Acad Sci USA. 2010;107(43):18664–18669. doi: 10.1073/pnas.1008734107. 20937863 PMC2972983

[6] Roenneberg T, Allebrandt KV, Merrow M, Vetter C. Social jetlag and obesity. Curr Biol CB. 2012;22(10):939–943. doi: 10.1016/j.cub.2012.03.038. 22578422

[7] Chakradeo PS, Keshavarzian A, Singh S, Dera AE, Esteban JPG, Lee AA, Burgess HJ, Fogg L, Swanson GR. Chronotype, social jet lag, sleep debt and food timing in inflammatory bowel disease. Sleep Med. 2018;52:188–195. doi: 10.1016/j.sleep.2018.08.002. 30243610 PMC8177729

[8] Asher G, Sassone-Corsi P. Time for food: the intimate interplay between nutrition, metabolism, and the circadian clock. Cell. 2015;161(1):84–92. doi: 10.1016/j.cell.2015.03.015. 25815987

[9] Bishehsari F, Engen PA, Voigt RM, Swanson G, Shaikh M, Wilber S, Naqib A, Green SJ, Shetuni B, Forsyth CB, et al. Abnormal eating patterns cause circadian disruption and promote alcohol-associated colon carcinogenesis. Cellular Mol Gastroenterol Hepatol. 2020;9(2):219–237. doi: 10.1016/j.jcmgh.2019.10.011. PMC6957855 31689559

[10] Zarrinpar A, Chaix A, Yooseph S, Panda S. Diet and feeding pattern affect the diurnal dynamics of the gut microbiome. Cell Metab. 2014;20(6):1006–1017. doi: 10.1016/j.cmet.2014.11.008. 25470548 PMC4255146

[11] Leone V, Gibbons SM, Martinez K, Hutchison AL, Huang EY, Cham CM, Pierre JF, Heneghan AF, Nadimpalli A, Hubert N, et al. Effects of diurnal variation of gut microbes and high-fat feeding on host circadian clock function and metabolism. Cell Host & Microbe. 2015;17(5):681–689. doi: 10.1016/j.chom.2015.03.006. 25891358 PMC4433408

[12] Swanson GR, Gorenz A, Shaikh M, Desai V, Kaminsky T, Van Den Berg J, Murphy T, Raeisi S, Fogg L, Vitaterna MH, et al. Night workers with circadian misalignment are susceptible to alcohol-induced intestinal hyperpermeability with social drinking. Am J Physiol. 2016;311(1):G192–G201. doi: 10.1152/ajpgi.00087.2016. PMC4967173 27198191

[13] Vancamelbeke M, Vermeire S. The intestinal barrier: a fundamental role in health and disease. Expert Rev Gastroenterol Hepatol. 2017;11(9):821–834. doi: 10.1080/17474124.2017.1343143. 28650209 PMC6104804

[14] Guo S, Al-Sadi R, Said HM, Ma TY. Lipopolysaccharide causes an increase in intestinal tight junction permeability in vitro and in vivo by inducing enterocyte membrane expression and localization of TLR-4 and CD14. Am J Pathol. 2013;182(2):375–387. doi: 10.1016/j.ajpath.2012.10.014. 23201091 PMC3562736

[15] Keshavarzian A, Fields JZ, Vaeth J, Holmes EW. The differing effects of acute and chronic alcohol on gastric and intestinal permeability. Am J Gastroenterol. 1994;89(12):2205–2211. 7977243

[16] Wang HJ, Zakhari S, Jung MK. Alcohol, inflammation, and gut-liver-brain interactions in tissue damage and disease development. World J Gastroenterol. 2010;16(11):1304–1313. doi: 10.3748/wjg.v16.i11.1304. 20238396 PMC2842521

[17] Frausto DM, Engen PA, Naqib A, Jackson A, Tran L, Green SJ, Shaikh M, Forsyth CB, Keshavarzian A, Voigt RM. Impact of alcohol-induced intestinal microbiota dysbiosis in a rodent model of Alzheimer’s disease. Front Aging. 2022;3:916336. doi: 10.3389/fragi.2022.916336. 36046496 PMC9421609

[18] Lu R, Voigt RM, Zhang Y, Kato I, Xia Y, Forsyth CB, Keshavarzian A, Sun J. Alcohol injury damages intestinal stem cells. Alcohol Clin Exp Res. 2017;41(4):727–734. doi: 10.1111/acer.13351. 28195397 PMC5378625

[19] Swanson GR, Garg K, Shaikh M, Keshavarzian A. Increased intestinal permeability and decreased resiliency of the intestinal barrier in alcoholic liver disease. Clin Transl Gastroenterol. 2024;15(4):e00689. doi: 10.14309/ctg.0000000000000689. 38334953 PMC11042778

[20] Jochum SB, Engen PA, Shaikh M, Naqib A, Wilber S, Raeisi S, Zhang L, Song S, Sanzo G, Chouhan V, et al. Colonic epithelial circadian disruption worsens dextran sulfate sodium-induced colitis. Inflamm Bowel Dis. 2023;29(3):444–457. doi: 10.1093/ibd/izac219. 36287037 PMC9977234

[21] Tran L, Jochum SB, Shaikh M, Wilber S, Zhang L, Hayden DM, Forsyth CB, Voigt RM, Bishehsari F, Keshavarzian A, et al. Circadian misalignment by environmental light/dark shifting causes circadian disruption in colon. PLOS ONE. 2021;16(6):e0251604. doi: 10.1371/journal.pone.0251604. 34086699 PMC8177509

[22] Summa KC, Voigt RM, Forsyth CB, Shaikh M, Cavanaugh K, Tang Y, Vitaterna MH, Song S, Turek FW, Keshavarzian A, et al. Disruption of the circadian clock in mice increases intestinal permeability and promotes alcohol-induced hepatic pathology and inflammation. PLOS ONE. 2013;8(6):e67102. doi: 10.1371/journal.pone.0067102. 23825629 PMC3688973

[23] Voigt RM, Summa KC, Forsyth CB, Green SJ, Engen P, Naqib A, Vitaterna MH, Turek FW, Keshavarzian A. The circadian clock mutation promotes intestinal dysbiosis. Alcohol Clin Exp Res. 2016;40(2):335–347. doi: 10.1111/acer.12943. 26842252 PMC4977829

[24] Zhen Y, Ge L, Xu Q, Hu L, Wei W, Huang J, Loor JJ, Yang Q, Wang M, Zhou P. Normal light-dark and short-light cycles regulate intestinal inflammation, circulating short-chain fatty acids and gut microbiota in Period2 gene knockout mice. Front Immunol. 2022;13:848248. doi: 10.3389/fimmu.2022.848248. 35371053 PMC8971677

[25] Wang S, Lin Y, Yuan X, Li F, Guo L, Wu B. REV-ERBα integrates colon clock with experimental colitis through regulation of NF-κB/NLRP3 axis. Nat Commun. 2018;9(1):4246. doi: 10.1038/s41467-018-06568-5. 30315268 PMC6185905

[26] Zhang Z, Li W, Han X, Tian D, Yan W, Liu M, Cao L. Circadian rhythm disruption-mediated downregulation of Bmal1 exacerbates DSS-induced colitis by impairing intestinal barrier. Front Immunol. 2024;15:1402395. doi: 10.3389/fimmu.2024.1402395. 38895112 PMC11183104

[27] Swanson GR, Siskin J, Gorenz A, Shaikh M, Raeisi S, Fogg L, Forsyth C, Keshavarzian A. Disrupted diurnal oscillation of gut-derived short chain fatty acids in shift workers drinking alcohol: possible mechanism for loss of resiliency of intestinal barrier in disrupted circadian host. Transl Res. 2020;221:97–109. doi: 10.1016/j.trsl.2020.04.004. 32376406 PMC8136245

[28] Davidson AJ, Poole AS, Yamazaki S, Menaker M. Is the food-entrainable circadian oscillator in the digestive system? Genes Brain Behav. 2003;2(1):32–39. doi: 10.1034/j.1601-183x.2003.00005.x. 12882317

[29] Hoogerwerf WA, Hellmich HL, Cornélissen G, Halberg F, Shahinian VB, Bostwick J, Savidge TC, Cassone VM. Clock gene expression in the murine gastrointestinal tract: endogenous rhythmicity and effects of a feeding regimen. Gastroenterology. 2007;133(4):1250–1260. doi: 10.1053/j.gastro.2007.07.009. 17919497

[30] Shaikh M, Rajan K, Forsyth CB, Voigt RM, Keshavarzian A. Simultaneous gas-chromatographic urinary measurement of sugar probes to assess intestinal permeability: use of time course analysis to optimize its use to assess regional gut permeability. Clinica (Rome) Acta; Int J Clin Chem. 2015;442:24–32. doi: 10.1016/j.cca.2014.12.040. PMC4339548 25591964

[31] Mutlu E, Keshavarzian A, Engen P, Forsyth CB, Sikaroodi M, Gillevet P. Intestinal dysbiosis: a possible mechanism of alcohol-induced endotoxemia and alcoholic steatohepatitis in rats. Alcohol Clin Exp Res. 2009;33(10):1836–1846. doi: 10.1111/j.1530-0277.2009.01022.x. 19645728 PMC3684271

[32] Deaver JA, Eum SY, Toborek M. Circadian disruption changes gut microbiome taxa and functional gene composition. Front Microbiol. 2018;9:737. doi: 10.3389/fmicb.2018.00737. 29706947 PMC5909328

[33] Fabregat A, Sidiropoulos K, Viteri G, Forner O, Marin-Garcia P, Arnau V, D’Eustachio P, Stein L, Hermjakob H. Reactome pathway analysis: a high-performance in-memory approach. BMC Bioinf. 2017;18(1):142. doi: 10.1186/s12859-017-1559-2. PMC5333408 28249561

[34] Neasta J, Darcq E, Jeanblanc J, Carnicella S, Ben Hamida S. GPCR and Alcohol-related behaviors in genetically modified mice. J Am Soc Exp Neurotherapeu. 2020;17(1):17–42. doi: 10.1007/s13311-019-00828-y. PMC7007453 31919661

[35] Wood S, Pithadia R, Rehman T, Zhang L, Plichta J, Radek KA, Forsyth C, Keshavarzian A, Shafikhani SH, Weber CR. Chronic alcohol exposure renders epithelial cells vulnerable to bacterial infection. PLOS ONE. 2013;8(1):e54646. doi: 10.1371/journal.pone.0054646. 23358457 PMC3554638

[36] Forsyth CB, Voigt RM, Shaikh M, Tang Y, Cederbaum AI, Turek FW, Keshavarzian A. Role for intestinal CYP2E1 in alcohol-induced circadian gene-mediated intestinal hyperpermeability. Am J Physiol. 2013;305(2):G185–G195. doi: 10.1152/ajpgi.00354.2012. PMC3725682 23660503

[37] Park JH, Jung IK, Lee Y, Jin S, Yun HJ, Kim BW, Kwon HJ. Alcohol stimulates the proliferation of mouse small intestinal epithelial cells via wnt signaling. Biochem Bioph Res Co. 2021;534:639–645. doi: 10.1016/j.bbrc.2020.11.028. 33220923

[38] Taleb Z, Carmona-Alcocer V, Stokes K, Haireek M, Wang H, Collins SM, Khan WI, Karpowicz P. BMAL1 regulates the daily timing of colitis. Front Cell Infect Microbiol. 2022;12:773413. doi: 10.3389/fcimb.2022.773413. 35223537 PMC8863668

[39] Yoo SH, Yamazaki S, Lowrey PL, Shimomura K, Ko CH, Buhr ED, Siepka SM, Hong HK, Oh WJ, Yoo OJ, et al. PERIOD2: LUCIFERASE real-time reporting of circadian dynamics reveals persistent circadian oscillations in mouse peripheral tissues. Proc Natl Acad Sci USA. 2004;101(15):5339–5346. doi: 10.1073/pnas.0308709101. 14963227 PMC397382

[40] Moore SR, Pruszka J, Vallance J, Aihara E, Matsuura T, Montrose MH, Shroyer NF, Hong CI. Robust circadian rhythms in organoid cultures from PERIOD2: LUCIFERASE mouse small intestine. Dis Model Mech. 2014;7(9):1123–1130. doi: 10.1242/dmm.014399. 24997189 PMC4142732

[41] Oh-Oka K, Kono H, Ishimaru K, Miyake K, Kubota T, Ogawa H, Okumura K, Shibata S, Nakao A, Weber CR. Expressions of tight junction proteins occludin and claudin-1 are under the circadian control in the mouse large intestine: implications in intestinal permeability and susceptibility to colitis. PLOS ONE. 2014;9(5):e98016. doi: 10.1371/journal.pone.0098016. 24845399 PMC4028230

[42] Forsyth CB, Shaikh M, Bishehsari F, Swanson G, Voigt RM, Dodiya H, Wilkinson P, Samelco B, Song S, Keshavarzian A. Alcohol feeding in mice promotes colonic hyperpermeability and changes in colonic organoid stem cell fate. Alcohol Clin Exp Res. 2017;41(12):2100–2113. doi: 10.1111/acer.13519. 28992396 PMC5711563

[43] Summa KC, Vitaterna MH, Turek FW, Ebihara S. Environmental perturbation of the circadian clock disrupts pregnancy in the mouse. PLOS ONE. 2012;7(5):e37668. doi: 10.1371/journal.pone.0037668. 22649550 PMC3359308

[44] Rodríguez-González A, Vitali F, Moya M, De Filippo C, Passani MB, Orio L. Effects of alcohol binge drinking and oleoylethanolamide pretreatment in the gut microbiota. Front Cell Infect Microbiol. 2021;11:731910. doi: 10.3389/fcimb.2021.731910. 34888256 PMC8651011

[45] Chen T, Kim CY, Kaur A, Lamothe L, Shaikh M, Keshavarzian A, Hamaker BR. Dietary fibre-based SCFA mixtures promote both protection and repair of intestinal epithelial barrier function in a caco-2 cell model. Food Function. 2017;8(3):1166–1173. doi: 10.1039/c6fo01532h. 28174773

[46] Laudisi F, Di Fusco D, Dinallo V, Stolfi C, Di Grazia A, Marafini I, Colantoni A, Ortenzi A, Alteri C, Guerrieri F, et al. The food additive maltodextrin promotes endoplasmic reticulum stress-driven mucus depletion and exacerbates intestinal inflammation. Cellular Mol Gastroenterol Hepatol. 2019;7(2):457–473. doi: 10.1016/j.jcmgh.2018.09.002. PMC6369223 30765332

[47] Ellacott KL, Morton GJ, Woods SC, Tso P, Schwartz MW. Assessment of feeding behavior in laboratory mice. Cell Metab. 2010;12(1):10–17. doi: 10.1016/j.cmet.2010.06.001. 20620991 PMC2916675

[48] Vogle A, Qian T, Zhu S, Burnett E, Fey H, Zhu Z, Keshavarzian A, Shaikh M, Hoshida Y, Kim M, et al. Restricted immunological and cellular pathways are shared by murine models of chronic alcohol consumption. Sci Rep. 2020;10(1):2451. doi: 10.1038/s41598-020-59188-9. 32051453 PMC7016184

[49] Bishehsari F, Preuss F, Mirbagheri SS, Zhang L, Shaikh M, Keshavarzian A. Interaction of alcohol with time of eating on markers of circadian dyssynchrony and colon tissue injury. Chemico-Biol Interact. 2020;325:109132. doi: 10.1016/j.cbi.2020.109132. PMC7315934 32437693

[50] Jeanblanc J, Rolland B, Gierski F, Martinetti MP, Naassila M. Animal models of binge drinking, current challenges to improve face validity. Neurosci Biobehav Rev. 2019;106:112–121. doi: 10.1016/j.neubiorev.2018.05.002. 29738795

[51] Rhodes JS, Best K, Belknap JK, Finn DA, Crabbe JC. Evaluation of a simple model of ethanol drinking to intoxication in C57BL/6J mice. Physiol Behav. 2005;84(1):53–63. doi: 10.1016/j.physbeh.2004.10.007. 15642607

[52] Caporaso JG, Lauber CL, Walters WA, Berg-Lyons D, Huntley J, Fierer N, Owens SM, Betley J, Fraser L, Bauer M, et al. Ultra-high-throughput microbial community analysis on the illumina HiSeq and MiSeq platforms. ISME J. 2012;6(8):1621–1624. doi: 10.1038/ismej.2012.8. 22402401 PMC3400413

[53] Naqib A, Poggi S, Wang W, Hyde M, Kunstman K, Green SJ. Making and sequencing heavily multiplexed, high-throughput 16S ribosomal RNA gene amplicon libraries using a flexible, two-stage PCR protocol. Methods Mol Biol. 2018;1783:149–169. doi: 10.1007/978-1-4939-7834-2_7. 29767361

[54] Zhang J, Kobert K, Flouri T, Stamatakis A. PEAR: a fast and accurate illumina paired-end reAd mergeR. Bioinf. 2014;30(5):614–620. doi: 10.1093/bioinformatics/btt593. PMC3933873 24142950

[55] Callahan BJ, McMurdie PJ, Rosen MJ, Han AW, Johnson AJ, Holmes SP. DADA2: High-resolution sample inference from Illumina amplicon data. Nat Methods. 2016;13(7):581–583. doi: 10.1038/nmeth.3869. 27214047 PMC4927377

[56] Estaki M, Jiang L, Bokulich NA, McDonald D, González A, Kosciolek T, Martino C, Zhu Q, Birmingham A, Vázquez-Baeza Y, et al. QIIME 2 enables comprehensive end-to-end analysis of diverse microbiome data and comparative studies with publicly available data. Curr Protocol Bioinformatics. 2020;70(1):e100. doi: 10.1002/cpbi.100. PMC9285460 32343490

[57] Quast C, Pruesse E, Yilmaz P, Gerken J, Schweer T, Yarza P, Peplies J, Glöckner FO. The SILVA ribosomal RNA gene database project: improved data processing and web-based tools. Nucleic Acids Res. 2013;41(Database issue):D590–D596. doi: 10.1093/nar/gks1219. 23193283 PMC3531112

[58] Bokulich NA, Kaehler BD, Rideout JR, Dillon M, Bolyen E, Knight R, Huttley GA, Gregory Caporaso J. Optimizing taxonomic classification of marker-gene amplicon sequences with QIIME 2‘s q2-feature-classifier plugin. Microbiome. 2018;6(1):90. doi: 10.1186/s40168-018-0470-z. 29773078 PMC5956843

[59] Davis NM, Proctor DM, Holmes SP, Relman DA, Callahan BJ. Simple statistical identification and removal of contaminant sequences in marker-gene and metagenomics data. Microbiome. 2018;6(1):226. doi: 10.1186/s40168-018-0605-2. 30558668 PMC6298009

[60] Hanshew AS, Mason CJ, Raffa KF, Currie CR. Minimization of chloroplast contamination in 16S rRNA gene pyrosequencing of insect herbivore bacterial communities. J Microbiol Methods. 2013;95(2):149–155. doi: 10.1016/j.mimet.2013.08.007. 23968645 PMC4133986

[61] Van der Sluis M, De Koning BA, De Bruijn AC, Velcich A, Meijerink JP, Van Goudoever JB, Büller HA, Dekker J, Van Seuningen I, Renes IB, et al. Muc2-deficient mice spontaneously develop colitis, indicating that MUC2 is critical for colonic protection. Gastroenterology. 2006;131(1):117–129. doi: 10.1053/j.gastro.2006.04.020. 16831596

[62] Sato T, Vries RG, Snippert HJ, van de Wetering M, Barker N, Stange DE, van Es JH, Abo A, Kujala P, Peters PJ, et al. Single Lgr5 stem cells build crypt-villus structures in vitro without a mesenchymal niche. Nature. 2009;459(7244):262–265. doi: 10.1038/nature07935. 19329995

[63] Sato T, Stange DE, Ferrante M, Vries RG, Van Es JH, Van den Brink S, Van Houdt WJ, Pronk A, Van Gorp J, Siersema PD, et al. Long-term expansion of epithelial organoids from human colon, adenoma, adenocarcinoma, and Barrett’s epithelium. Gastroenterology. 2011;141(5):1762–1772. doi: 10.1053/j.gastro.2011.07.050. 21889923

[64] Jung P, Sato T, Merlos-Suárez A, Barriga FM, Iglesias M, Rossell D, Auer H, Gallardo M, Blasco MA, Sancho E, et al. Isolation and in vitro expansion of human colonic stem cells. Nat Med. 2011;17(10):1225–1227. doi: 10.1038/nm.2470. 21892181

[65] Co JY, Margalef-Català M, Li X, Mah AT, Kuo CJ, Monack DM, Amieva MR. Controlling epithelial polarity: a human enteroid Model for host-pathogen interactions. Cell Rep. 2019;26(9):2509–2520.e4. doi: 10.1016/j.celrep.2019.01.108. 30811997 PMC6391775

[66] Sun L, Jia H, Li J, Yu M, Yang Y, Tian D, Zhang H, Zou Z. Cecal gut microbiota and metabolites might contribute to the severity of acute myocardial ischemia by impacting the intestinal permeability, oxidative stress, and energy metabolism. Front Microbiol. 2019;10:1745. doi: 10.3389/fmicb.2019.01745. 31428065 PMC6687875

[67] Metabolon. Experimental procedures. n.d. [accessed 2023 Jan 11]. https://www.metabolon.com/software/support/portal/experimental-procedures/.

[68] Ford L, Kennedy AD, Goodman KD, Pappan KL, Evans AM, Miller LAD, Wulff JE, Wiggs BR, Lennon JJ, Elsea S, et al. Precision of a clinical metabolomics profiling platform for use in the identification of inborn errors of metabolism. J Appl Lab Med. 2020;5(2):342–356. doi: 10.1093/jalm/jfz026. 32445384

[69] Erben U, Loddenkemper C, Doerfel K, Spieckermann S, Haller D, Heimesaat MM, Zeitz M, Siegmund B, Kühl AA. A guide to histomorphological evaluation of intestinal inflammation in mouse models. Int J Clin Exp Pathol. 2014;7(8):4557–4576. 25197329 PMC4152019

[70] Ruxton GD, Beauchamp G. Time for some a priori thinking about post hoc testing. Behavioral Ecol. 2008;19(3):690–693. doi: 10.1093/beheco/arn020.

[71] R: the R project for statistical computing. https://www.r-project.org/.

[72] Kelly BJ, Gross R, Bittinger K, Sherrill-Mix S, Lewis JD, Collman RG, Bushman FD, Li H. Power and sample-size estimation for microbiome studies using pairwise distances and PERMANOVA. Bioinformatics. 2015;31(15):2461–2468. doi: 10.1093/bioinformatics/btv183. 25819674 PMC4514928

[73] Anderson MJ. Distance-based tests for homogeneity of multivariate dispersions. Biometrics. 2006;62(1):245–253. doi: 10.1111/j.1541-0420.2005.00440.x. 16542252

[74] Quinn TP, Erb I, Gloor G, Notredame C, Richardson MF, Crowley TM. A field guide for the compositional analysis of any-omics data. GigaScience. 2019;8(9):giz107. doi: 10.1093/gigascience/giz107. 31544212 PMC6755255

[75] Li Y, Andrade J. Deapp: an interactive web interface for differential expression analysis of next generation sequence data. Source Code Biol Med. 2017;12(1):2. doi: 10.1186/s13029-017-0063-4. 28174599 PMC5291987

[76] Love MI, Huber W, Anders S. Moderated estimation of Fold change and dispersion for RNA-seq data with DESeq2. Genome Biol. 2014;15(12):550. doi: 10.1186/s13059-014-0550-8. 25516281 PMC4302049

[77] Weiss S, Xu ZZ, Peddada S, Amir A, Bittinger K, Gonzalez A, Lozupone C, Zaneveld JR, Vázquez-Baeza Y, Birmingham A, et al. Normalization and microbial differential abundance strategies depend upon data characteristics. Microbiome. 2017;5(1):27. doi: 10.1186/s40168-017-0237-y. 28253908 PMC5335496

[78] Gillespie M, Jassal B, Stephan R, Milacic M, Rothfels K, Senff-Ribeiro A, Griss J, Sevilla C, Matthews L, Gong C, et al. The reactome pathway knowledgebase 2022. Nucleic Acids Res. 2022;50(D1):D687–D692. doi: 10.1093/nar/gkab1028. 34788843 PMC8689983

[79] Zielinski T, Moore AM, Troup E, Halliday KJ, Millar AJ, Yamazaki S. Strengths and limitations of period estimation methods for circadian data. PLOS ONE. 2014;9(5):e96462. doi: 10.1371/journal.pone.0096462. 24809473 PMC4014635

[80] Cornelissen G. Cosinor-based rhythmometry. Theor Biol Med Model. 2014;11(1):16. doi: 10.1186/1742-4682-11-16. 24725531 PMC3991883

[81] Nelson W, Tong YL, Lee JK, Halberg F. Methods for cosinor-rhythmometry. Chronobiologia. 1979;6(4):305–323. 548245

[82] Izumo M, Pejchal M, Schook AC, Lange RP, Walisser JA, Sato TR, Wang X, Bradfield CA, Takahashi JS. Differential effects of light and feeding on circadian organization of peripheral clocks in a forebrain Bmal1 mutant. eLife. 2014;3:e04617. doi: 10.7554/eLife.04617. 25525750 PMC4298698

