# Supplementary material for: Impact of peripheral circadian misalignment and alcohol on the resiliency of intestinal barrier and microbiota
Source: Gut Microbes. 2025 Jun 4;17(1):2509281. doi: 10.1080/19490976.2025.2509281 (PMC12143687; doi:10.1080/19490976.2025.2509281)
Supplement: Supporting_Documents_for_Food_Timing_Alcohol_Manuscript.docx [file KGMI_A_2509281_SM8979.docx]

Supporting Documents (In order of appearance)

##### **Impact of Wrong Time Eating (WTE) and Alcohol on Food/Liquid Consumption and Weight in Mice**

Average weekly food and liquid intake were significantly affected by treatment and sex effects. Males consumed more food, as expected, but consumed less liquid compared to female mice. Body weight was assessed and was significantly affected by alcohol treatment and sex effects. Male mice were more affected by alcohol treatment, resulting in decreased average body weight. Average weekly alcohol intake was measured and there was a significant impact of the food timing paradigm on alcohol consumption (Supplementary Figure 1).

##### **Impact of Wrong Time Eating (WTE) on Alcohol Consumption in Mice**

The blood alcohol levels were assessed with significantly elevated alcohol effects noted in the right time eating alcohol-fed group (RTE EtOH), consistent with blood collected following 12 hours of food intake and 20% EtOH (v/v) availability. It is significantly increased compared to the H₂O groups. However, there was not a significantly elevated blood alcohol in the wrong-time alcohol-fed (WTE EtOH) group due to a study constraint. The time of sacrifice occurred in the morning although we anticipate this trend to have continued with significantly increased levels if this group was sacrificed at night when the mice are coming off 12 hours of food intake and 20% EtOH (v/v) availability (F).


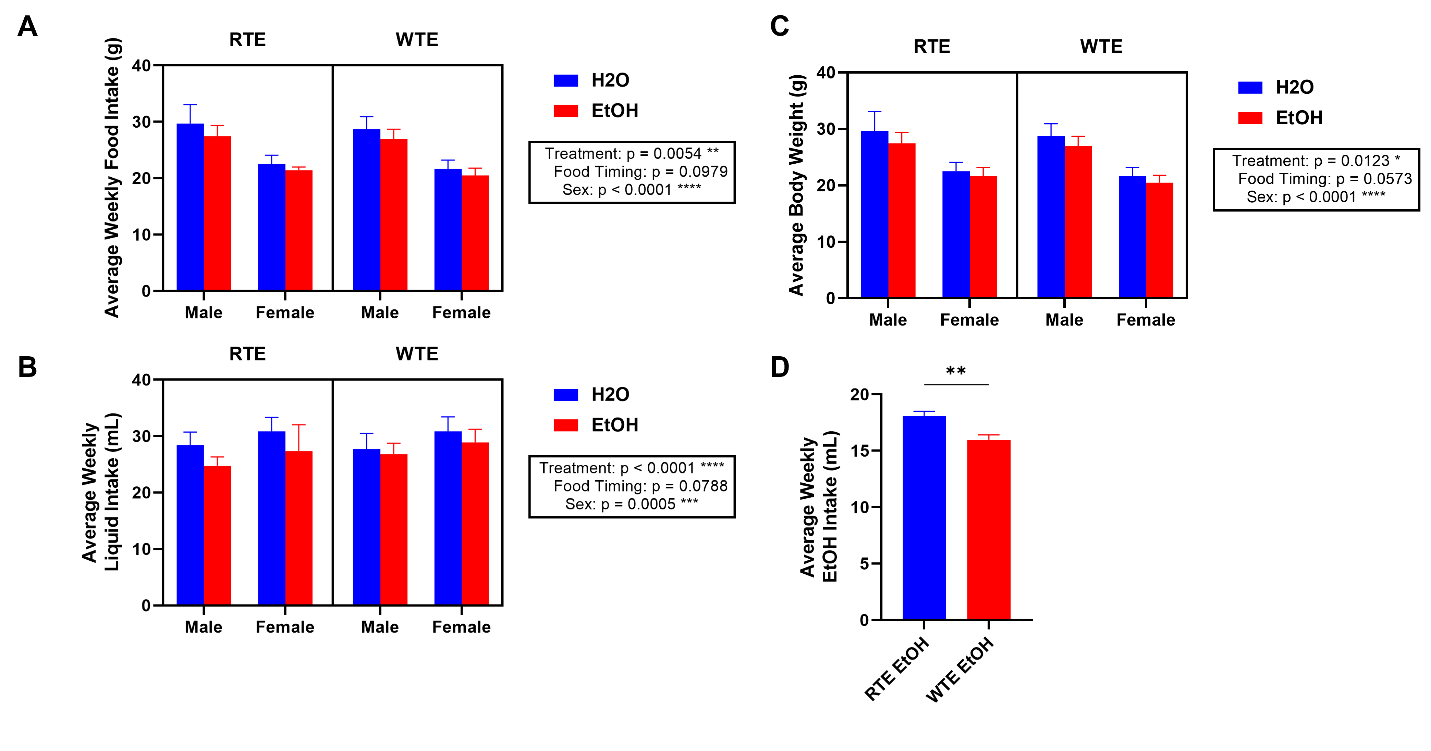


Supplemental Figure 1. Average weekly food and liquid intake. (A) Average weekly food intake is impacted by both alcohol treatment and sex. (B) Average weekly overall liquid intake (H₂O and EtOH) is significantly impacted by alcohol treatment and sex. (C) Average body weight is impacted by both alcohol treatment and sex. Female mice are unaffected by food timing and alcohol treatment. (D) Average alcohol intake was significantly affected by altered food timing. Two- and Three-way ANOVA (results in box) were conducted, and effects are indicated on each graph when significant: ^*^p < 0.05, ^∗∗^p < 0.01, ^∗∗∗^p < 0.001, and ^∗∗∗∗^p < 0.0001.


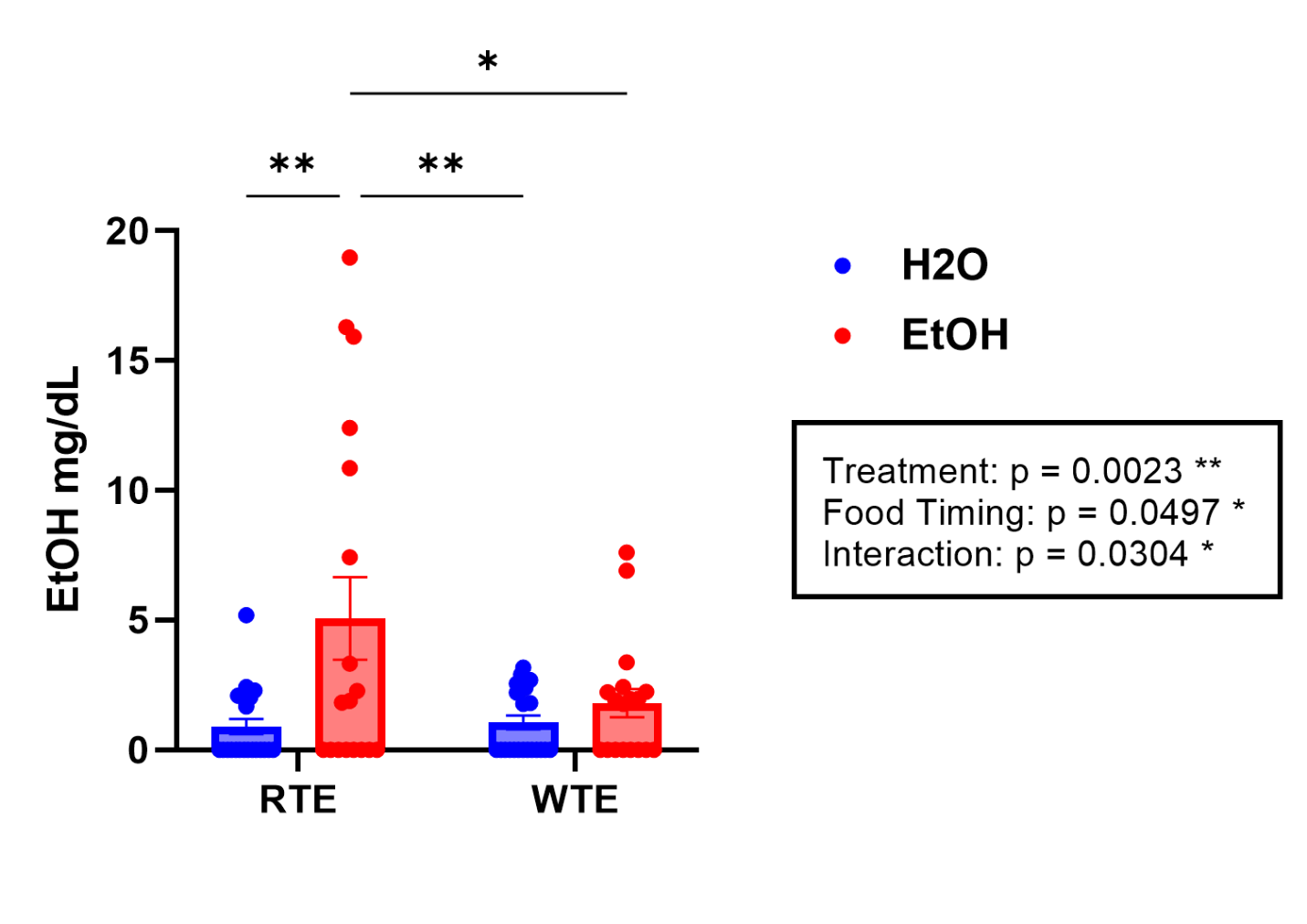


Supplemental Figure 2. Blood alcohol content in mice. Serum blood alcohol was measured and analyzed. ETOH (mg/dL) was significantly increased in RTE ETOH groups. WTE EtOH blood alcohol levels were not significant due to study constraints. Between n = 18-23 mice/treatment group. Two-way ANOVA (results in box) was conducted, and effects are indicated on each graph when significant: ^*^p < 0.05 and ^∗∗^p < 0.01.

#### Supplemental Table 1. Histology score comparisons in all mouse groups.


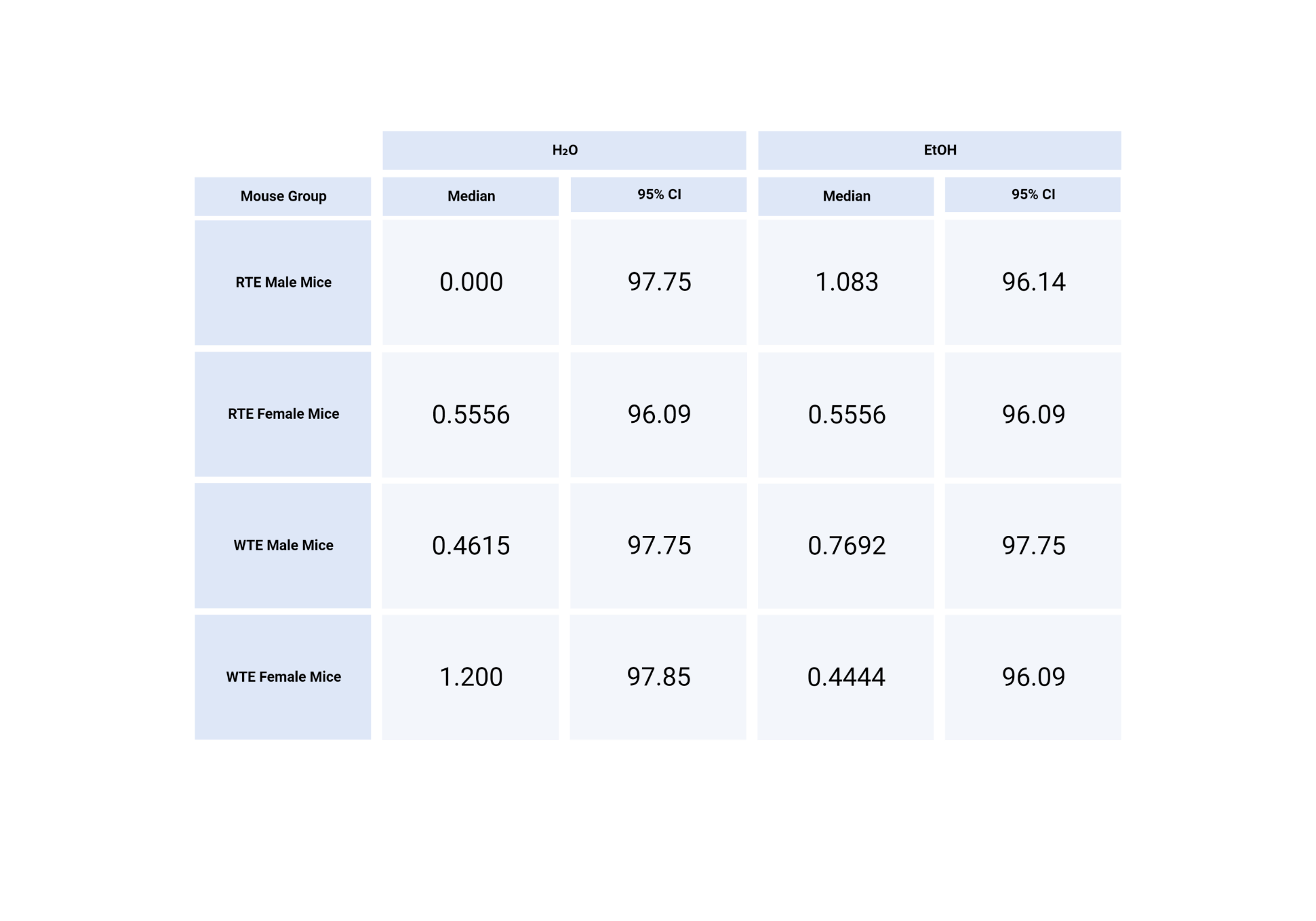


Abbreviations: CI, confidence interval; EtOH, ethanol alcohol; H₂O, water; RTE, right time eating; WTE, wrong time eating

**
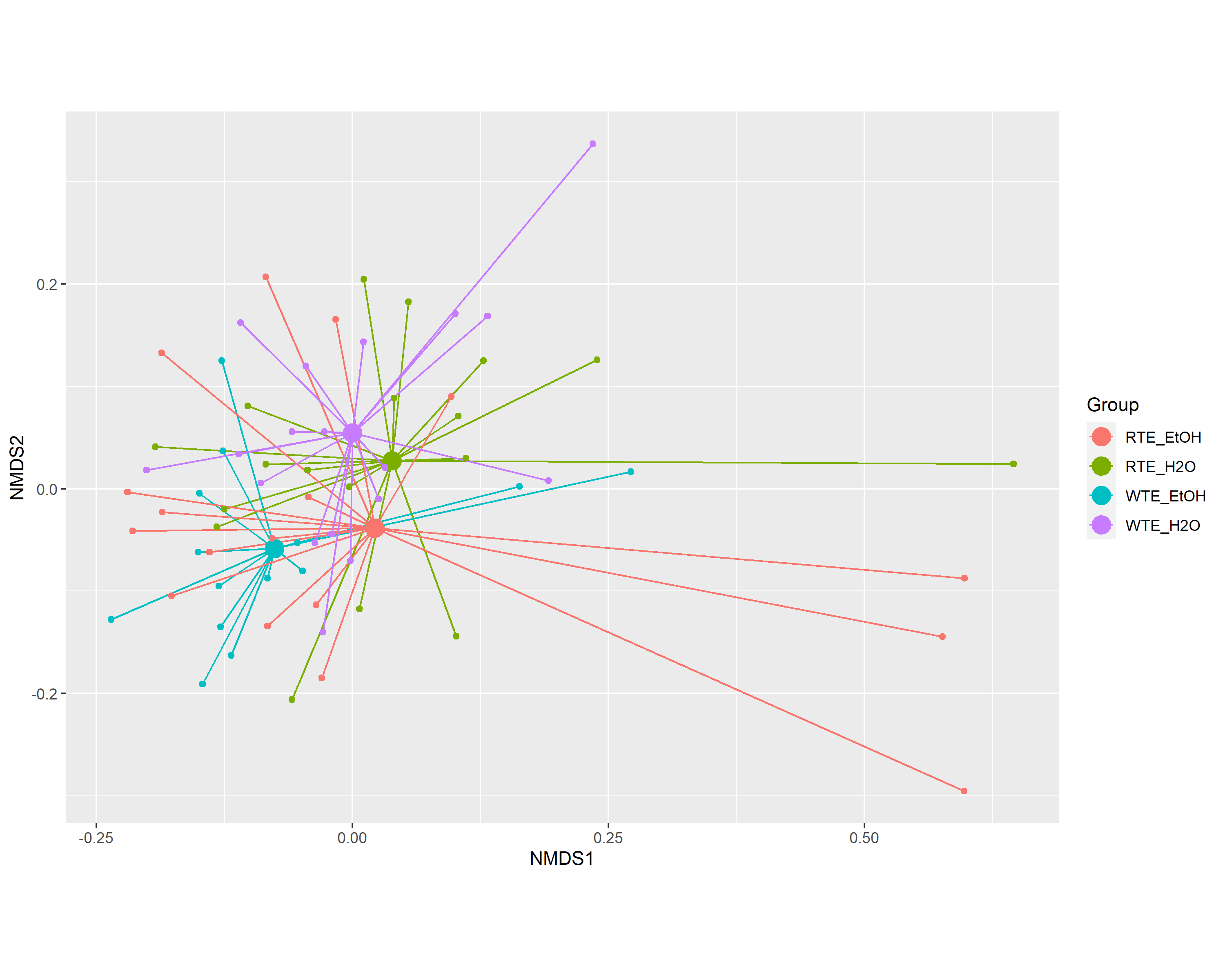
**

Supplemental Figure 3. Non-metric multidimensional scaling (NMDS) of all mouse groups.


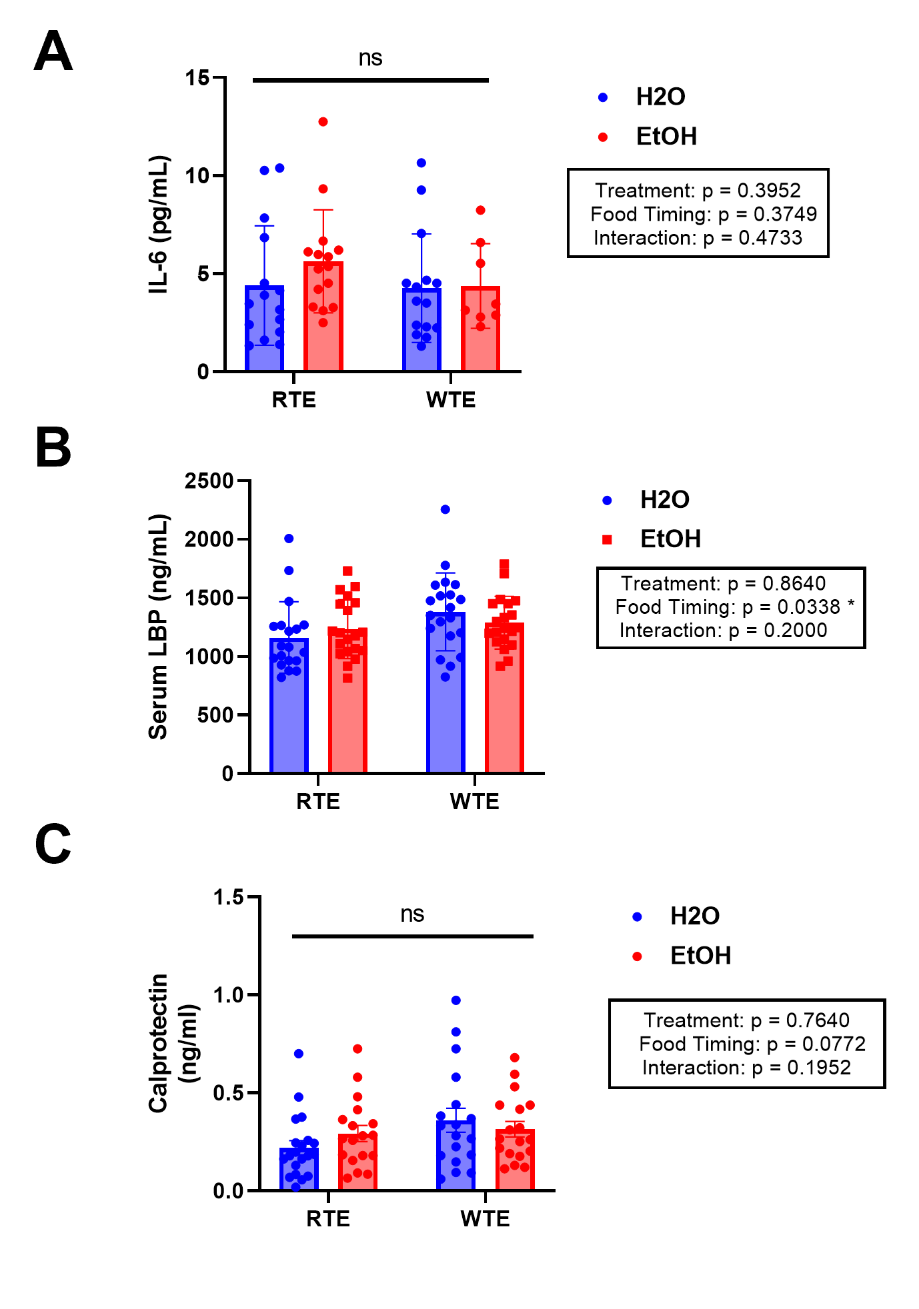


Supplemental Figure 4. Effect of altered food timing and alcohol consumption on inflammatory markers in mice. (A) Serum cytokine IL-6 levels were measured and analyzed. IL-6 (pg/ml) was not impacted by the genotype or alcohol consumption nor was there an interaction. Between n = 8-17 mice/treatment group. (B) Serum LBP exhibited a significant effect of food timing but no interaction. Between n = 19-20 mice/treatment group. (C) Stool calprotectin levels were measured but no significant effects were observed. Between n = 18-20 mice/treatment group. Two-way ANOVA (results in box) was conducted, and effects are indicated on each graph when significant: ^*^p < 0.05.


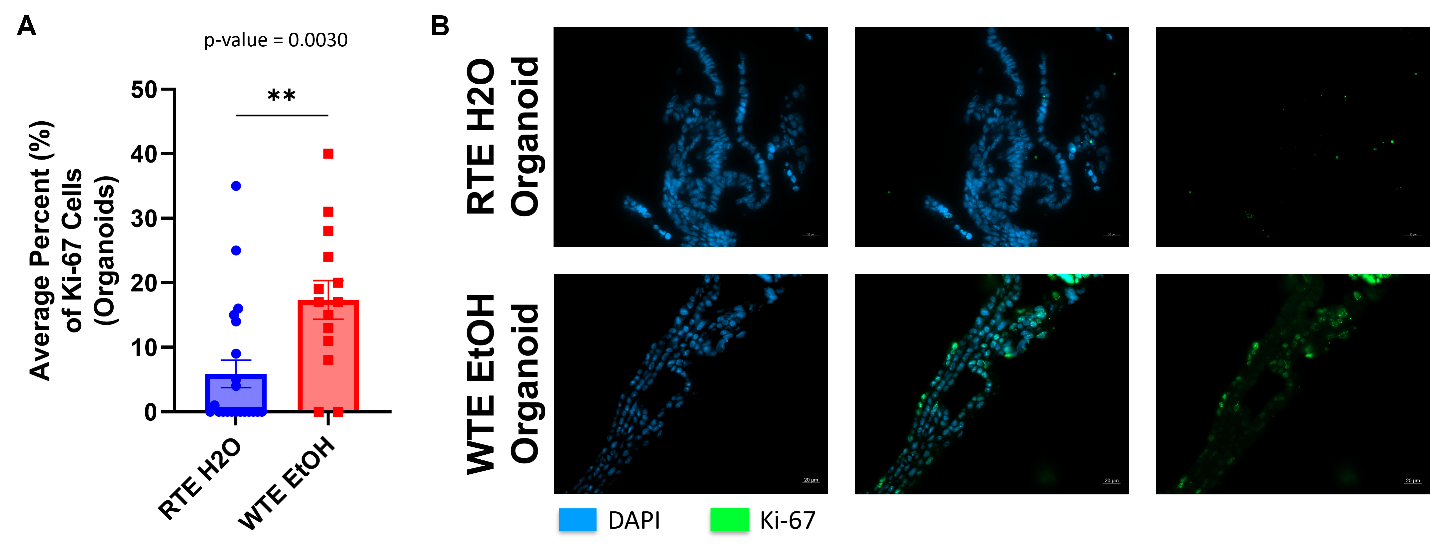

Supplemental Figure 5. Increased proliferative activity of Ki-67 in WTE EtOH organoids. (A) Intestinal organoids derived from WTE EtOH mice had significantly increased levels of Ki-67, a marker of cell proliferation. (B) Immunofluorescent staining of Ki-67 in organoids showed an increase of Ki-67 in the WTE EtOH organoids. All images were taken at ×40 magnification. Roughly 300 organoids per group were analyzed and an average percentage of Ki-67 present was calculated. P-values are indicated on each graph when significant:  ^∗∗^p < 0.01.


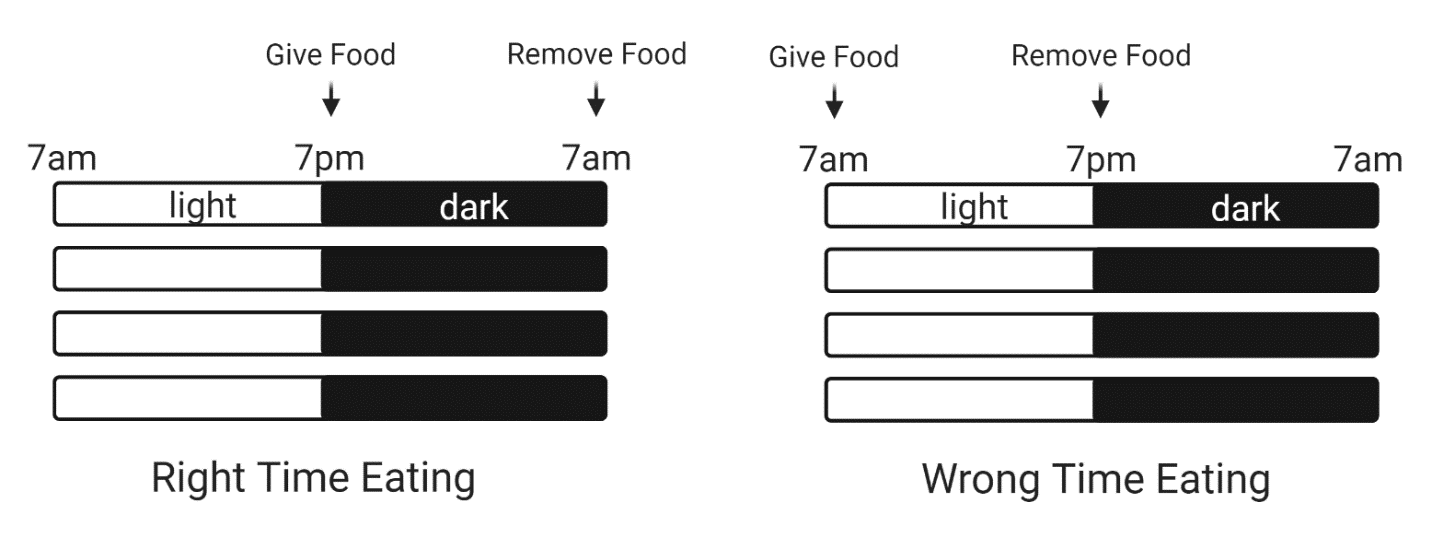


Supplemental Figure 6. Food timing paradigm. Right-time eating (RTE) is defined as feeding during the biological active phase. As mice are nocturnal, the RTE is 7 pm. Wrong-time eating (WTE) is defined as feeding during the biological rest phase where feeding occurs at 7 am. Mice are given chow for a total of 12 hours, with alcohol coinciding with food intake. Conditions are ad-libitum on the weekend to mimic human conditions. Created with BioRender.com


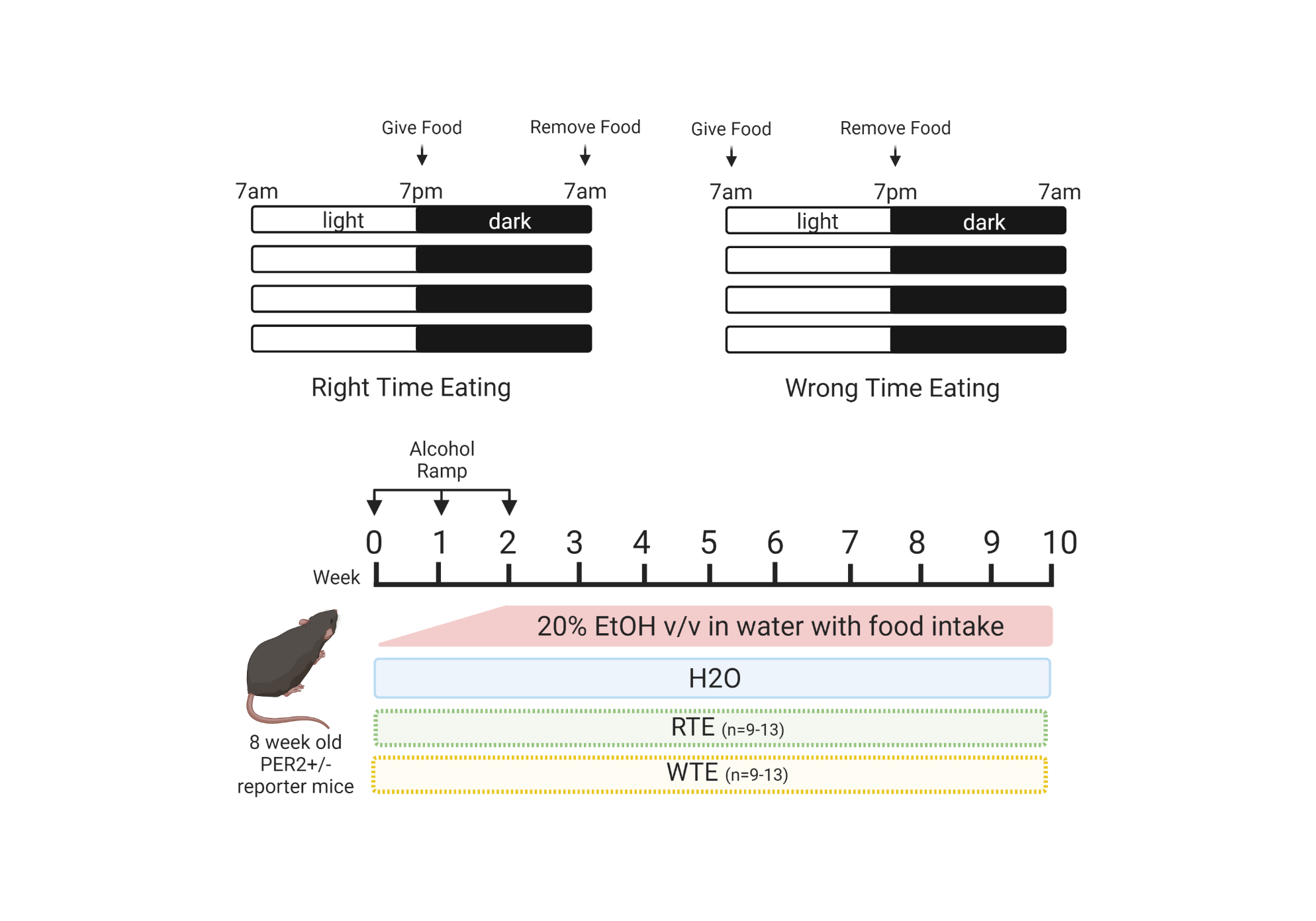


Supplemental Figure 7. Overall study timeline. PER2::LUC mice were placed in one of four groups (RTE H₂O, RTE EtOH, WTE H₂O, and WTE EtOH). Those in alcohol groups received an increasing ramp of alcohol concentrations before being given 20% EtOH (v/v) in drinking water with food intake. The study lasted 10 weeks with weekly mouse weight and stool collection occurring and daily liquid consumption (H₂O and EtOH) was measured. Tissue and blood collection occurred at the end of the study during the sacrifice. Created with BioRender.com

Supplemental Table 2. List of primary antibodies for immunofluorescent staining.

Supplemental Table 3. List of primary antibodies for immunoblotting.
